# Fabrication of Bi_2_O_3_/Bismuth Titanates Modified with Metal–Organic Framework-In_2_S_3_/CdIn_2_S_4_ Materials for Electrocatalytic H_2_ Production and Its Photoactivity

**DOI:** 10.1021/acs.langmuir.3c02031

**Published:** 2023-10-16

**Authors:** Krishnakumar Balu, Balakrishna Avula, Mani Durai, Sakthivel Kumaravel, Ernesto Chicardi, Ranier Sepúlveda, Elangovan Erusappan, Imran Hasan, Young-Ho Ahn

**Affiliations:** † Departamento de Ingeniería y Ciencia de los Materiales y del Transporte, E.T.S. de Ingenieros, Universidad de Sevilla, Avda. Camino de los Descubrimientos s/n., 41092 Sevilla, Spain; ‡ Department of Chemistry, Rajeev Gandhi Memorial College of Engineering and Technology (Autonomous), Nandyal, Andhra Pradesh 518501, India; § Environmental Science and Engineering Laboratory, Department of Civil Engineering, Yeungnam University, Gyeongsan 38541, Republic of Korea; ∥ Department of Environmental Engineering, Korea Maritime and Ocean University, Busan 49112, Republic of Korea; ⊥ Department of Applied Science and Technology, Anna University, Chennai, Tamil Nadu 600025, India; # Department of Chemistry, College of Science, King Saud University, Riyadh 11451, Saudi Arabia; ∇ Department of Chemistry, Saveetha School of Engineering, Saveetha Institute of Medical and Technical Sciences, Saveetha University, Chennai, Tamil Nadu 602105, India

## Abstract

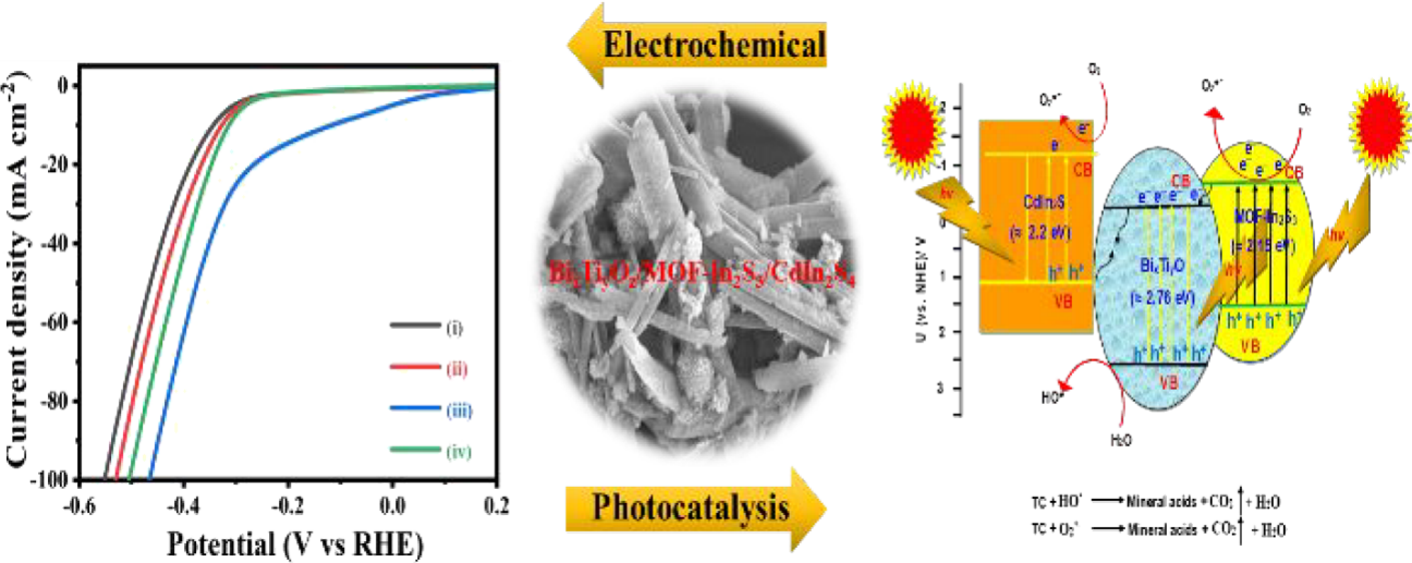

Compositional and structural elucidation of the materials is important to know their properties, chemical stability, and electro-photoactivity. The heterojunction electrocatalyst and photocatalyst activity could open a new window for solving the most urgent environmental and energy problems. Here, for the first time, we have designed and fabricated Bi_2_O_3_/bismuth titanates modified with MOF-In_2_S_3_/CdIn_2_S_4_ materials by a stepwise process. The detailed structural elucidation and formation of mixed composite phases were studied in detail. It has been found that the formed composite was efficiently utilized for the electrocatalytic H_2_ production reaction and the photocatalytic degradation of tetracycline. XRD patterns for the metal–organic framework-In_2_S_3_ showed a main compound of MOF, and it was assigned to a MIL-53 MOF phase, with a monoclinic structure. The addition of CdCl_2_ onto the MOF-In_2_S_3_ phase effectively produced a CdIn_2_S_4_ flower platform on the MOF rods. The uniform dispersion of the bismuth titanates in MOF-In_2_S_3_/CdIn_2_S_4_ materials is detected by mapping of elements obtained by dark-field HAADF-STEM. Finally, the predictions of how to integrate experiments and obtain structural results more effectively and their common development in new heterojunctions for electro-/photocatalytic applications are presented.

## Introduction

Currently, global energy production is majorly dependent on fossil fuels. Industrialization energy needs to scale up as a function of population growth; it has been expected that the current energy demand will be increased three times in the next few decades, and more clean energy sources such as hydrogen need to be used. To date, a large number of composite materials have been reported for efficient hydrogen evolution and harmful compound degradation.^1−12^ However, from the application point of view, it is still difficult to utilize these materials for efficient production of H_2_ using direct solar light due to the wide band gap (>3 eV), being only active under high energy UV light. Bi_2_O_3_-based materials are potential electrocatalytic materials with high conductivity and low melting points.^13,14^ Therefore, at the beginning of the 21st century, researchers focused on bismuth-based materials for energy and environmental-related applications.^15−17^

Due to the close relationship between the above constraints and their active characteristics, the adjustment of size, shape, and crystal structure is essentially dynamic.^18−22^ The practical applications in several fields make the synthesis of modified binary and ternary chalcogenide compounds by various methods extremely interesting for research. Due to their distinct optoelectronic, electronic, optical, and catalytic capabilities, metal sulfides and chalcogenides (ternary, AB_2_S_4_; A = M^2+^, B = M^3+^) have been the focus of significant research for several years.^23−27^ Due to its intriguing applications in the disciplines of optics, photoconductivity, and optoelectronics, In_2_S_3_, a binary chalcogenide, has garnered special attention. Its high photoconductivity, chemical stability, and low-dimensional or nanoscale size made In_2_S_3_ a promising material for redox catalysts.^28−30^ It also has good electron transport properties and slow charge recombination, which make it a suitable catalyst for photo- and electrocatalytic applications.

Sheng-qi Guo et al.^31^ developed a self-assembly method for the hydrothermal production of In_2_S_3_ nanotubes along with rGO. The process is performed through the use of InCl_3_, thioacetamide, and GO as precursors and poly(vinyl alcohol) (PVA) as a template in a water medium. The authors claimed that the sulfur source and template are important parameters for directing the structure and morphology of the produced In_2_S_3_. The formed composite was effectively utilized for electrocatalytic iodide species reduction reaction. Efficient photoelectrochemical (PEC) water splitting via Zr-doped In_2_S_3_ 2D nanoflakes was reported by Ligang Wang et al.^32^ The nanoflakes were obtained by reaction between In(NO_3_)_3_.4H_2_O and thioacetamide in an octanol/octylamine mixture via a hydrothermal approach. The Zr-modified In_2_S_3_ has relatively negative conduction band edges and favors the visible light absorbance tendency via the comfortable band gap energies.

Because of their numerous industrial applications, AB_2_S_4_-type semiconductors with layered structures have gained some attention. Recently, Xi Chen et al.^33^ created a globular flower-like CuS/CdIn_2_S_4_/ZnIn_2_S_4_ and successfully used it for hydrogen evaluation and harmful pollutant degradation processes. The composite material was synthesized using nitrate salts of metal ion (Cu, Cd, In, and Zn) precursors with thioacetamide by a microwave-assisted hydrothermal reaction. The surface morphology of the produced composite was globular and flower-like, which contributed significantly to its high surface area and catalytic activity. Metal–organic frameworks (MOFs) have received a lot of interest recently because of their adaptable structural design, significant specific surface area, and high porosity.^34^ MOF-based CdIn_2_S_4_ (CdIn_2_S_4_@NH_2_-MIL-125) was synthesized via the hydrothermal method.^34^ Authors claimed that the efficient production of hydrogen by the composite may be due to the formation of a heterojunction of NH_2_-MIL-125 with CdIn_2_S_4_.

Even pristine or unmodified bismuth oxides have good catalytic properties. Bismuth-based nanocomposites have incredible applications in wastewater treatment and electrochemical and energy production.^35−39^ There are many bismuth-based composites available in the literature; out of the reported developed materials, the bismuth titanates have been shown as efficient base materials for energy and environmental applications. Ning Zhang^40^ reported that the photocatalytic activity of Bi_4_Ti_3_O_12_ increased when modified with CuS. The combination of these materials was obtained by hydrothermal synthesis. The modified composite showed potential degradation activity against Rhodamine B (Rh B) dye. Another bismuth titanate-based material Bi_12_TiO_20_ modified with hydrochar and BiOBr was reported by Yulan Ren.^41^ The formed HC/BiOBr/Bi_12_TiO_20_ microsphere is efficiently utilized for pollutant degradation. Here, we report the formation of the bismuth oxide/bismuth titanate phase modified with MOF/In_2_S_3_. The addition of cadmium ions produced CdIn_2_S_4_ via an ion-exchange reaction on the surface of MOF/In_2_S_3._ The structural and morphological properties and the combination of the composite materials were studied in detail. The combination of bismuth titanates with MOF-In_2_S_4_/CdIn_2_S_4_ is effectively utilized for electrocatalytic “H_2_” evaluation reactions. In addition, the photocatalytic activity against tetracycline (TC) degradation under solar light has been studied.

## Materials and Methods

### Chemicals Used for This Study

Cadmium Chloride (CdCl_2_·5H_2_O), indium nitrate In(NO_3_)_3_·*X*H_2_O, bismuth nitrate (Bi(NO_3_)_3_·5H_2_O) pentahydrate, 1,4 benzene carboxylic acid, titanium isopropoxide, antibiotic TC, thiourea, dimethylformamide (DMF), benzyl alcohol, bought from Sigma-Aldrich, served as the basis for creating the bismuth oxide/bismuth titanates/MOF-In_2_S_4_/CdIn_2_S_4_ composites.

### Instrumentations

The crystallinity and the structural properties of the MOF-In_2_S_4_, MOF-In_2_S_4_/CdIn_2_S_4_, and bismuth titanates have been determined by powder X-ray diffraction patterns utilizing the Miniflex, Rigaku, Japan, PXRD [nickel filter CuKα radiation (30 kV, = 1.5419; range of 10–80)] system. The visible absorbance and band gap of the synthesized materials were obtained by a Neosys-2000 instrument (UV-DRS, in the range between 200 and 800 nm). The photoluminescent measurements were made with a Scinco (Korea) model spectrofluorometer. The surface morphology of the materials was obtained (10 kV- accelerating voltage) by a Hitachi S-4800 instrument (FE-SEM). On a Si-wafer substrate, the samples were coated. With a 120 kV operating accelerating voltage, the FEI-Tecnai TF-20 transmission electron microscope was used to create TEM/FE-TEM pictures and selected area electron diffraction (SAED) patterns. Before the tests were performed, the samples were dispersed in ethanol and put onto copper grids coated with carbon. With the use of K-Alpha from Thermo Scientific, XPS spectra were obtained. The main excitation was the nonmonochromatic Al K line at 180–200 W. Using Micromeritics ASAP 2000 and nitrogen adsorption at 77 K, the specific surface areas of the materials were calculated using the BET equation.

### Synthesis of Bismuth Titanates (Bi_*x*_Ti_*y*_O_*z*_)

About 3.64 g (7.5 × 10^–3^ M) of bismuth nitrate pentahydrate was dissolved in 15 mL of benzyl alcohol and stirred for 15 min. To this was added 15 mL of benzyl alcoholic solution containing 0.186 g (0.646 × 10^–3^ M) of titanium isopropoxide. The stirring was continued for 4 h. After that, the mixture was transferred into a 50 mL of stainless-steel Teflon autoclave and kept at 130 °C for 24 h. The final precipitate was obtained after three washings with a (1:1) EtOH:H_2_O solution, 48 h of drying at 80 °C, and 3 h of calcination in a muffle furnace at 450 °C.

### MIL- 53 (In) Prism (MOF)

A 20 mL solution of DMF containing 1100 mg of 1,4-benzene carboxylic acid (Terephthalic acid) was added, while stirring, to the 900 mg of indium nitrate that had been dissolved in the 20 mL of DMF. The mixture was then reflexed for 30 min at 120 °C. Three EtOH washes were performed on the precipitate that had developed (MOF).

### Synthesis of In_2_S_3_ from the MIL- 53 Indium Precursor (MOF-In_2_S_3_)

The obtained MIL- 53 prism was added into 40 mL of an ethanolic solution containing 685 mg of thiourea under stirring conditions, and stirring continued further for 15 min. It was then put into a 50 mL Teflon-coated stainless-steel autoclave and kept at 180 °C for 3h. The formed precipitate (MOF-In_2_S_3_) was washed three times with the mixture of (1:1) EtOH:H_2_O and dried at 60 °C for 5 h.

### Synthesis of MOF-In_2_S_3_/CdIn_2_S_4_

About 0.8145 g of MOF-In_2_S_3_ (0.0025 M with respect to In_2_S_4_) was dispersed in 20 mL of water, then 20 mL of an aqueous solution containing 0.2854 g of (0.0012 M) CdCl_2_ was added while stirring at 60 °C, and then, stirring was continued further 10 min; after that, the formed precipitate was washed with ethanol and dried in at 80 °C for 24 h in order to obtained MOF-In_2_S_3_/CdIn_2_S_4_.

### Synthesis of Bi_*x*_Ti_*y*_O_*z*_/MOF-In_2_S_3_/CdIn_2_S_4_ and Bi_*x*_Ti_*y*_O_*z*_/MOF-In_2_S_3_

For Bi_*x*_Ti_*y*_O_*z*_/MOF-In_2_S_3_/CdIn_2_S_4,_ about 750 mg of Bi_*x*_Ti_*y*_O_*z*_ was dispersed in 25 mL of the 1:1 EtOH/water mixture. To this was added 25 mL of solution containing 250 mg of MOF-In_2_S_3_/CdIn_2_S_4_ dispersion. The stirring continued for 5h. The mixture was then filtered, and ethanol was washed and dried at 80 °C for 24 h. For comparison purposes, the composite Bi_*x*_Ti_*y*_O_*z*_/MOF-In_2_S_3_ was prepared with the same procedure with the respective amount of MOF-In_2_S_4_.

### Electrocatalytic Water Splitting Experiment

Linear sweep voltammetry (LSV) and cyclic voltammogram (CV) measurements were used to evaluate the H_2_ evaluation activity of the produced materials. The working electrodes were prepared by the following method. The samples were coated on nickel form (NiF) support (1 cm × 1 cm). About 10 mg of the samples was mixed with 10% polyvinylidene fluoride and 10% activated carbon in *N*-methyl-2-pyrrolidone. The sample-coated nickel form was dried at 50 °C overnight. Calomel electrodes (Hg/HgO electrodes) and platinum electrodes served as the counter and reference electrodes, respectively. The stable overlap curves were confirmed by multiple scanning CV obtained by 1 M KOH solution. The Tafel slopes were obtained using the LSV curve, and the overlap potential was set at 10 mA cm^–2^. The stability of the Bi_*x*_Ti_*y*_O_*z*_/MOF-In_2_S_3_/CdIn_2_S_4_/NiF electrode was confirmed by a chronopotentiometry study. For electrocatalytic water splitting, the IVIUM instrument was used.

### Photocatalytic Degradation Experiments under Solar Light

The produced materials’ photocatalytic abilities to degrade TC were assessed in direct sunlight (95 mW/cm^2^). The solar experiments were carried out in Gyeongsan, South Korea, between 11 a.m. and 2 p.m. The solar light intensity was nearly constant during the studies. In each experiment, 100 mL of 20 ppm TC solution was mixed with a measured quantity (20 mg/100 mL) of the synthesized materials in a beaker, and the dark reaction was performed under dark conditions for 1 h; in order to obtain the adsorption capability of the materials, after reaching the equilibrium, the catalyst containing solution was irradiated under direct sunlight. The samples (3–4 mL) were taken at predetermined intervals, centrifuged, and examined with a UV–vis spectrometer, and the analytical wavelength of TC was 357 nm.

## Results and Discussion

### XRD Analysis

**Figure 1:**
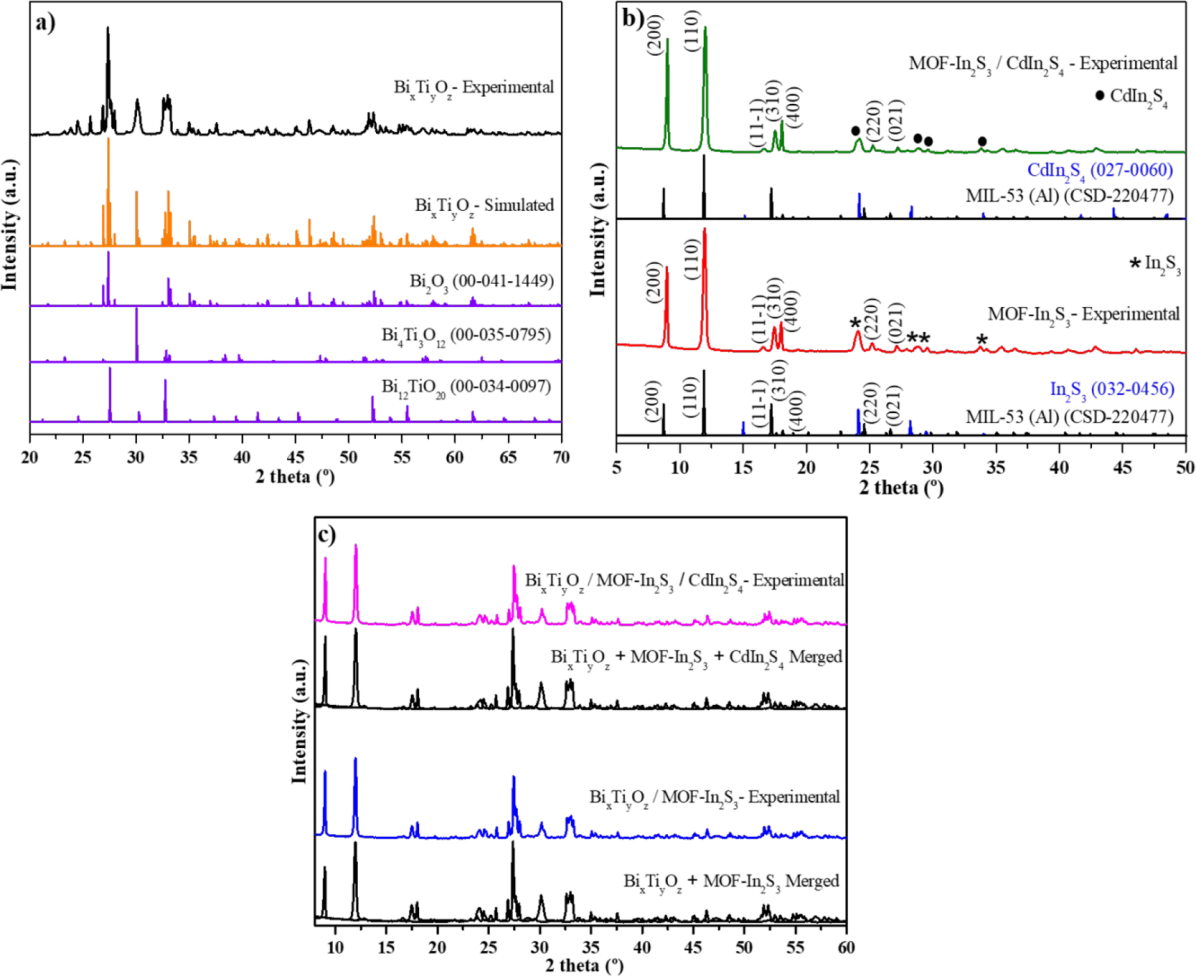
X-ray diffractograms for (a) Bi_*x*_Ti_*y*_O_*z*_ material (black) and the simulated XRD pattern (orange) from the indexed phases (blue); (b) MOF-In_2_S_3_ and MOF-In_2_S_3_/CdIn_2_S_4_ materials (red and green, respectively) together with the simulated XRDs for indexed phases (black and blue diffractograms); (c) Bi_*x*_Ti_*y*_O_*z*_ /MOF-In_2_S_3_ and Bi_*x*_Ti_*y*_O_*z*_ /MOF-In_2_S_3_/CdIn_2_S_4_ materials. Merged diffractograms from the experimentally synthesized materials are also displayed for comparison purposes.

The experimental XRD spectra for Bi_*x*_Ti_*y*_O_*z*_, MOF-In_2_S_3,_ Bi_*x*_Ti_*y*_O_*z*_/MOF-In_2_S_3_, Bi_*x*_Ti_*y*_O_*z*_/MOF-In_2_S_3_/CdIn_2_S_4,_ and MOF-In_2_S_3_/CdIn_2_S_4_ specimens are shown in Figure 1. Concretely, for the specimen Bi_*x*_Ti_*y*_O_*z*_ (Bi–Ti–O photocatalyst), the corresponding experimental XRD pattern (Figure 1a) showed the presence of a majority phase, bismuth oxide (Bi_2_O_3_), with a monoclinic structure and *P*21/*c* space group symmetry (SGS, ref no. 00-041-1449 in the PDF4 + ICDD database). Another two important phases could be also indexed: concretely, two different bismuth titanates. They are Bi_12_TiO_20_ (ref no. 00-034-0097, cubic structure, *I*23 SGS, in th- PDF4 + ICDD database) and Bi_4_Ti_3_O_12_ (ref no. 00-035-0795, orthorhombic structure, *Cmmm* SGS, in the PDF4 + ICDD database). The simulated XRD pattern from the three phases indexed (orange XRD in Figure 1a) matched with the experimental XRD, corroborating that these phases formed.

On the other hand, the specimen MOF-In_2_S_3_, corresponding to the experimental XRD pattern for the metal–organic framwork-In_2_S_3_ photocatalyst system (Figure 1b), showed a main compound that was assigned to a MIL-53 MOF phase, with a monoclinic structure and *Cc* SGS, according to reference no. 220477 found in the Cambridge Structural Database (CSD).

There, the cationic metallic element in the MOF showed is Al^3+^. However, for the MOF-In_2_S_3_ material, the metallic element corresponds to the In^3+^ cation. This aspect was caused due to the different cation radii tetracoordinate of Al^3+^ (0.39 Å) and In^3+^ (0.62 Å), a shift of peaks to lower 2 theta degree, as can be easily observed for the (200) and (110) crystallographic planes. Thus, MOF-53 (In) was correctly formed. Also, following the same CSD database, the indium sulfide (In_2_S_3_), with a cubic structure and *Fd*3̅*m* SGS (ref no 1596369 in the CSD database). Thus, no other phase could be detected in this MOF-In_2_S_3_ specimen, suggesting the successful formation of the MOF-In_2_S_3_ hybrid material.

Subsequently, the experimental XRD for the specimen with the addition of CdCl_2_ salt in the MOF-In_2_S_3_ specimen, according to the procedure mentioned above in the experimental section, revealed the presence of the MOF-53 (In)-In_2_S_3_ hybrid material; there was no decomposition during the procedure of introduction of CdCl_2_. In addition, another phase was indexed with the same structure and lattice parameter as the cubic In_2_S_3_. However, in this case, this phase corresponds to the ternary chalcogenide cadmium indium sulfide, CdIn_2_S_4_, according to the ref pattern no. 027-0060 of the PDF4 + -ICDD database (cubic structure and *Fd*3̅*m* SGS). As can be observed in the specimen labeled as MOF-In_2_S_3_/CdIn_2_S_4_ (see Figure 1b), this phase showed an analogous structure compared to indium sulfide.

Finally, in Figure 1c, the other two specimens developed are exposed, based on a combination of Bi_*x*_Ti_*y*_O_*z*_ and MOF-In_2_S_3_ (specimen Bi_*x*_Ti_*y*_O_*z*_/MOF-In_2_S_3_) and the mixture of Bi_*x*_Ti_*y*_O_*z*_ and MOF-In_2_S_3_/CdIn_2_S_4_ (specimen Bi_*x*_Ti_*y*_O_*z*_/MOF-In_2_S_3_/CdIn_2_S_4_). Thus, due to the procedure evolving only the suspension mixture in water, the phases formed are the summary of the existing phases of their reagents, as can be easily corroborated by attending to the analogous experimental XRD obtained by the merging of Bi_*x*_Ti_*y*_O_*z*_ with MOF-In_2_S_3_ and Bi_*x*_Ti_*y*_O_*z*_ with MOF-In_2_S_3_/CdIn_2_S_4_. Therefore, the indexed phases in Bi_*x*_Ti_*y*_O_*z*_/MOF-In_2_S_3_ are the above-mentioned Bi_*x*_Ti_*y*_O_*z*_ (Bi_2_O_3_, Bi_4_Ti_3_O_12_, and Bi_12_TiO_20_) and MOF-53 (In)-In_2_S_3_ from MOF-In_2_S_3_. Analogously, the specimen Bi_*x*_Ti_*y*_O_*z*_/MOF-In_2_S_3_/CdIn_2_S_4_ (Figure 1c) presented the same phases in addition to CdIn_2_S_4_. All these assertions extracted by XRD are corroborated later by the TEM analysis.

### Surface Functional Group Analysis and Optical Studies

**Figure 2:**
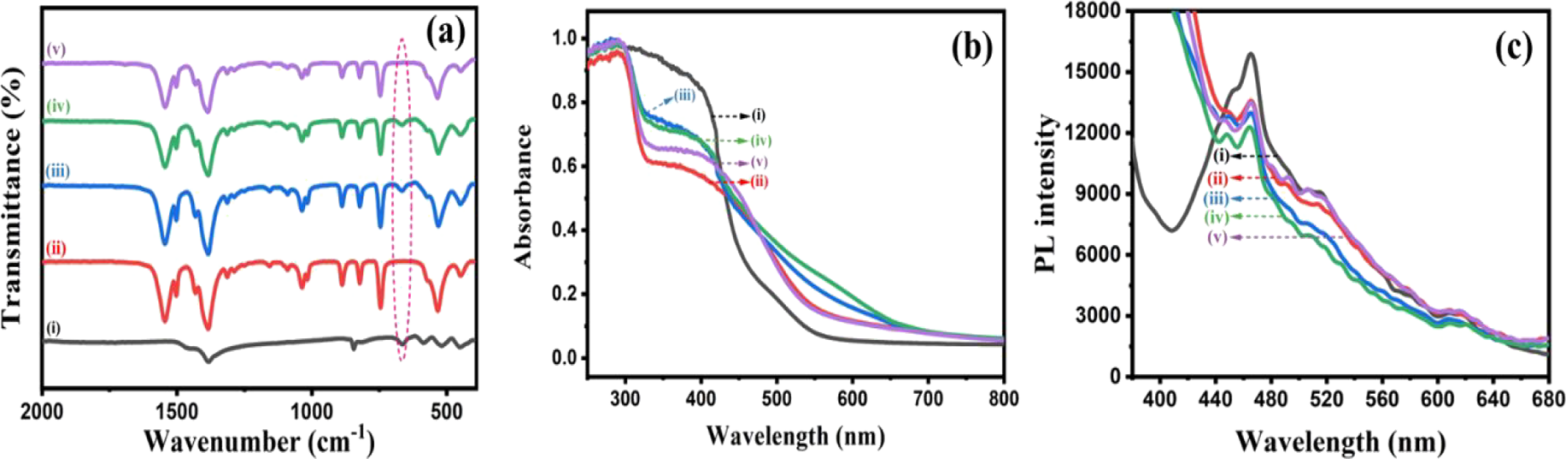
(a) FT-IR, (b) DRS, and (c) PL spectra of synthesized materials (i) Bi_*x*_Ti_*y*_O_*z*_, (ii) MOF-In_2_S_3_, (iii) Bi_*x*_Ti_*y*_O_*z*_/MOF-In_2_S_3_, (iv) Bi_*x*_Ti_*y*_O_*z*_/MOF-In_2_S_3_/CdIn_2_S_4_, and (v) MOF-In_2_S_3_/CdIn_2_S_4_.

The surface functional groups were analyzed by the IR technique, and the IR spectra of Bi_*x*_Ti_*y*_O_*z*_, MOF-In_2_S_3,_ Bi_*x*_Ti_*y*_O_*z*_/MOF-In_2_S_3_, Bi_*x*_Ti_*y*_O_*z*_/MOF-In_2_S_3_/CdIn_2_S_4,_ and MOF-In_2_S_3_/CdIn_2_S_4_ specimens are presented in Figure 2a. In Bi_*x*_Ti_*y*_O_*z*_ and its modified specimens, the common signal of oxygen–metal bonds is observed from 500 to 860 cm^–1^. The two significant peaks at about 846 and 586 cm^–1^ were attributed to the stretching vibrations of Bi–O and Ti–O bonds, respectively. This confirms the formation of the Bi_*x*_Ti_*y*_O_*z*_ structure.^42^ Moreover, in the Bi_*x*_Ti_*y*_O_*z*_ specimen, the strong bismuth titanate peak appeared at 657 cm^–1^, and this peak remains unchanged in all Bi_*x*_Ti_*y*_O_*z-*_containing specimens (Figure 2a, highlighted part). The IR spectrum of MOF-In_2_S_3_ and MOF-based composites showed that two strong bands that appeared at 1386 and 1548 cm^–1^ are assigned to asymmetric and symmetric stretching vibrations of O=C–O group (from Terephthalic acid) from MIL-53 MOF.^43^ The C=C groups in the benzene ring’s characteristic vibration are represented by the tiny band at 1501 cm^–1^, and C–H bending vibration is observed at 744 cm^–1^.^44^ The main characteristic absorption peak of In_2_S_3_ is observed at 1430 cm^–1^ for all MOF-In_2_S_3_ and MOF-loaded samples.^45^ After CdCl_2_ addition to MOF-In_2_S_3_, the IR spectrum of MOF-In_2_S_3_/CdIn_2_S_4_ almost resembles that of MOF-In_2_S_3_.

The light absorbance behavior of the prepared samples was measured via solid-state UV-DRS measurements, and the results are presented in Figure 2b. The DRS reveals that the Bi_*x*_Ti_*y*_Oz component has strong UV–vis absorbance until 430 nm, and after that, the absorbance intensity decreased in the entire visible region. The absorbance of MOF-In_2_S_3_ increased from 450 nm to the entire visible region (blue, 445–500 nm; green, 500–545 nm; 545- 580 nm, orange, 580–600 nm, and red-600–750 nm). The combination of these two composites shows a higher absorbance in the visible region from blue to red (445–750 nm); in particular, the visible absorbance of the Bi_*x*_Ti_*y*_O_*z*_/MOF-In_2_S_3_/CdIn_2_S_4_ component increased in the entire visible region when compared with other components. After the addition of CdCl_2_ into Bi_*x*_Ti_*y*_O_*z*_/MOF-In_2_S_3_, the absorbance intensity of Bi_*x*_Ti_*y*_O_*z*_/MOF-In_2_S_3_ increased from mid of blue to (480 nm) to red (750 nm) region; this may be the formation of CdIn_2_S_4_ phase, that is, Bi_*x*_Ti_*y*_O_*z*_/MOF-In_2_S_3_/CdIn_2_S_4_. The formed heterostructure (Bi_*x*_Ti_*y*_O_*z*_/MOF-In_2_S_3_/CdIn_2_S_4_) efficiently degrades the TC under direct solar light (discussion comes later) and effectively produced H_2_ from water (discussion comes later). This study clearly revealed that there was a close interaction observed between Bi_*x*_Ti_*y*_O_*z*_ and MOF-In_2_S_3,_ and CdIn_2_S_4_ components in Bi_*x*_Ti_*y*_O_*z*_/MOF-In_2_S_3_/CdIn_2_S_4_. After the addition of CdCl_2_ in the MOF-In_2_S_3_, the formation of a new phased occurred on the surface of MOF-In_2_S_3_ via the ion-exchange reaction and could absorb the light over a wide spectrum of solar light (from blue to red region), which enhanced the visible light activity of the Bi_*x*_Ti_*y*_O_*z*_/MOF-In_2_S_3_/CdIn_2_S_4_ component.

Figure 2c represents the photoluminescent properties of the synthesized specimens. The intensity of the Bi_*x*_Ti_*y*_O_*z*_ specimen is higher than those of all of the synthesized materials. Generally, the higher PL intensity is caused by the fast e^–^/h^+^ recombination, which does not favor photocatalytic applications. However, the PL intensity of the Bi_*x*_Ti_*y*_O_*z*_/MOF-In_2_S_3_/CdIn_2_S_4_ specimen is low compared with other components. The Bi_*x*_Ti_*y*_O_*z*_ specimen has the PL emission at 464 nm, and the other Bi_*x*_Ti_*y*_O_*z*_-based specimens have the same PL emission, but the intensities of the emissions are less when compared with the Bi_*x*_Ti_*y*_O_*z*_ specimen. It may be due to the suppression of e^–^/h^+^ recombination, which favors the photocatalytic applications.

### Surface Morphology Analysis by FE-SEM, TEM, and Elemental Mapping Studies

**Figure 3:**
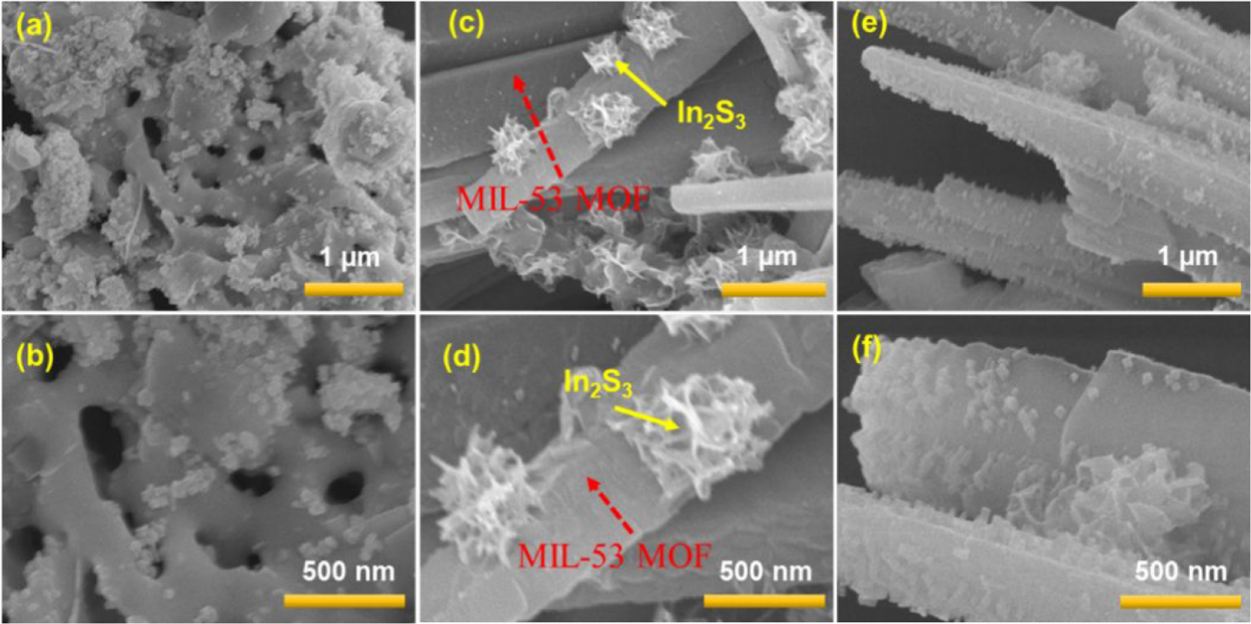
FE-SEM images of Bi_*x*_Ti_*y*_O_*z*_ (a and b), MOF-In_2_S_3_ (c and d), and Bi_*x*_Ti_*y*_O_*z*_/MOF-In_2_S_3_/CdIn_2_S_4_ (e and f).

Surface morphology of the synthesized materials initially observed by field emission scanning electron microscopy (FE-SEM) and FE-SEM images of Bi_*x*_Ti_*y*_O_*z*_, MOF-In_2_S_3,_ and Bi_*x*_Ti_*y*_O_*z*_/MOF-In_2_S_3_/CdIn_2_S_4_ are shown in Figures 3 and S1 (see Supporting Information). Bi_*x*_Ti_*y*_O_*z*_ has two different phases, well-separated round-shaped particles and shapeless layered structured particles (Figures 3a,b and S1a). In the same way, MOF-In_2_S_3_ also has two phases rod-like MIL-53 MOF and flower-like In_2_S_3_ (Figures 3c,d and S1b,c). The combination of these two particles produced Bi_*x*_Ti_*y*_O_*z*_/MOF-In_2_S_3_ (Figure S2a–c). The addition of Cd ion into this mixture produced Bi_*x*_Ti_*y*_O_*z*_/MOF-In_2_S_3_/CdIn_2_S_4_ (Figure 3e,f) and shows the individual Bi_*x*_Ti_*y*_O_*z*_ and MOF-In_2_S_3_ particles along with numerous small particles on the surface of MOF. This has been confirmed with MOF-In_2_S_3_/CdIn_2_S_4_ images (Figure S2d–f). The morphology and composition of the mixture in the composites were further analyzed by HR-TEM and elemental mapping analysis, and the results are discussed in detail.

**Figure 4:**
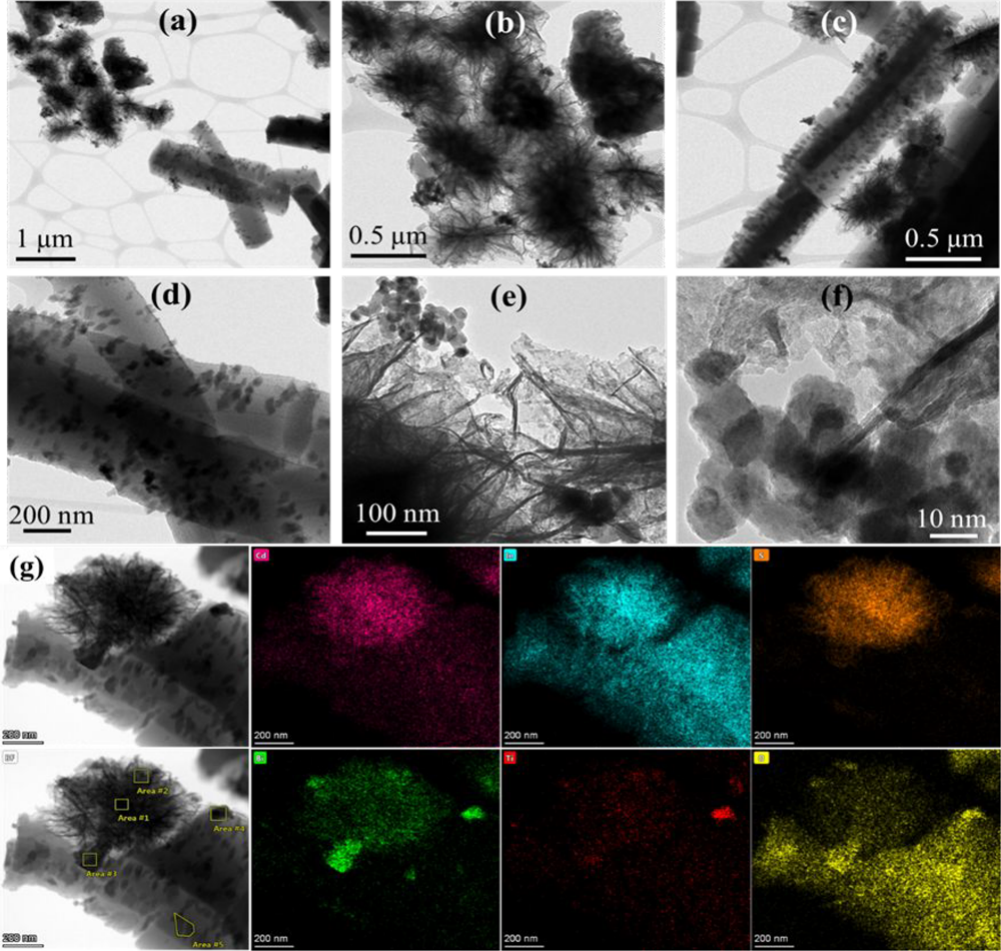
HR-TEM images (a–f) and the corresponding mapping (g) of the Bi_*x*_Ti_*y*_O_*z*_/MOF-In_2_S_3_/CdIn_2_S_4_ material. Colors in mapping: pink = Cd; light blue = In; orange = S; green = Bi; red = Ti; and yellow = O.

The TEM study was carried out on the Bi_*x*_Ti_*y*_O_*z*_/MOF-In_2_S_3_/CdIn_2_S_4_ specimen, and the corresponding images with various locations and magnifications are shown in Figure 4. This specimen has been selected to corroborate the indexed phases formed because it is the most complex specimen, formed by the mixture of the specimens Bi_*x*_Ti_*y*_O_*z*_ and MOF-In_2_S_3_/CdIn_2_S_4_. The particles detected in Figure 4 exhibit different and diverse morphologies. Concretely, it was observed the typical irregular flower-type architectures were widely reported for the CdIn_2_S_4._^46−48^

The architecture is, in fact, agglomerated with nanoparticles, as can be observed in Figure 4f. This morphology is not a strange structure. These irregular flower-type architectures are also commonly used for other complex sulfides like ZnIn_2_S_4_^49,50^ and, even, for cubic β-In_2_S_3_^51,52^ synthesized in the specimen MOF-In_2_S_3_, as the reagent to obtain the Bi_*x*_Ti_*y*_O_*z*_/MOF-In_2_S_3_/CdIn_2_S_4_ specimen. In addition, mapping XEDS carried out on different flower types of CdIn_2_S_4_ particles (areas 1 and 2 in Figure 4g, areas 2 and 3 in Figure S3, and area 1 in Figure S4) corroborated the presence of Cn, In, and S in them. Thus, the punctual semiquantitative XEDS composition gave the average composition displayed in Table 1, according to the CdIn_2_S_4_ stoichiometry.

**Table 1:** Punctual Semiquantitative XEDS Composition of the Bi_*x*_Ti_*y*_O_*z*_/MOF-In_2_S_3_/CdIn_2_S_4_ Specimen

phase	elements					
	**Bi**	**Cd**	**In**	**O**	**S**	**Ti**
CdIn_2_S_4_		11.2 ± 1.9	30.9 ± 2.1		58.0 ± 0.9	
MOF-53 (In)			19.5 ± 2.0	71.5 ± 2.0		
In_2_O_3_			36.5 ± 1.5	63.5 ± 1.5		
Bi–Ti–O	17.9 ± 2.8			67.9 ± 2.6		14.2 ± 0.5
Bi_2_O_3_	67.2 ± 1.8			32.8 ± 2.2		-

In Figure 4, the other particles with different morphologies revealed submicrometric rods that are acting as hosts for embedded nanoparticles. By the analogous mapping XEDS study carried out for the flower-type CdIn_2_S_4_, it was observed that these rods are mainly composed of In and O, suggesting that they are, in fact, the MOF-53 (In) indexed by XRD (see Figures 4g, S3, and S4). Also, the embedded nanoparticles showed higher In and O brightness in comparison to the rest of the rod. In addition, the embedded nanoparticles are composed of indium oxide (In_2_O_3_), as shown in the marked area 5 in Figure 4g, which was not previously detected by XRD due to its nanometric size and low amount, due to which it can be masked by the rest of phases. Then, the submicrometer rods are formed, in fact, by In_2_O_3_@MOF-53. This type of hybrid material has been already developed by other systems like Au/MO*x*@MOF-5 (M = Zn, Ti; *x* = 1,2), Ru@MOF-5, and so forth.^53^

**Figure 5:**
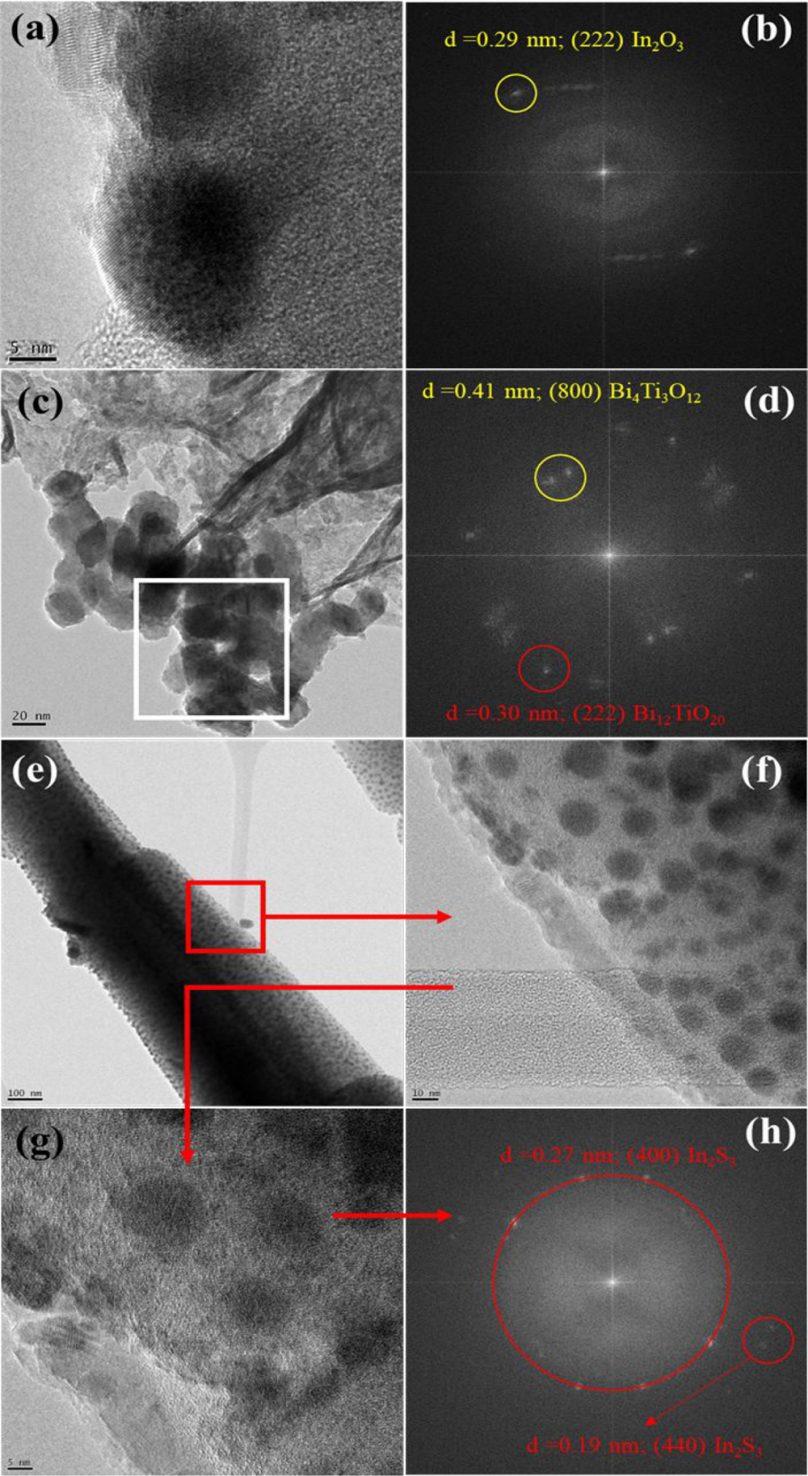
(a–d) HRTEM and FFT images for the Bi_*x*_Ti_*y*_O_*z*_/MOF-In_2_S_3_/CdIn_2_S_4_ material. The image (b) corresponds to the FFT image for the full HRTEM image displayed in (a). Image (d) shows the FFT for the white square marked in image (c); (e–h) HRTEM and FFT images for the Bi_*x*_Ti_*y*_O_*z*_/MOF-In_2_S_3_ material. The image (h) corresponds to the FFT image of the red square of image (g).

In addition, by HRTEM (Figure 5a) and the corresponding FFT (Figure 5b) images of those nanoparticles could be indexed, the interplanar distance of the (222) crystallographic planes (*d* = 0.29 nm) could be indexed, corroborating the In_2_O_3_ nature of those nanoparticles. However, the presence of In_2_O_3_ is interesting since the XRD for Bi_*x*_Ti_*y*_O_*z*_/MOF-In_2_S_3_ specimens used as mixing is formed by In_2_S_3_ instead of In_2_O_3_. Thus, the introduction of Cd in Bi_*x*_Ti_*y*_O_*z*_/MOF-In_2_S_3_/CdIn_2_S_4_ caused the transformation of some In_2_S_3_ by In_2_O_3_ because of the excess amount of S required, and the remaining excess In^3+^ cations react with oxygen to form nanoembedded particles of In_2_O_3_ into the MOF rods. To clarify this aspect, HRTEM images have been obtained also in the Bi_*x*_Ti_*y*_O_*z*_/MOF-In_2_S_3_ specimen. In Figure 5e–h, a high number of embedded nanoparticles could be clearly observed, corresponding undoubtfully to In_2_S_3_. The transformation of In_2_S_3_ in In_2_O_3_ did not take place due to the absence of Cd, which prevents the breakdown of In_2_S_3_. The indexation of the (400) and (440) interplanar distance from the embedded nanoparticles in the Bi_*x*_Ti_*y*_O_*z*_/MOF-In_2_S_3_ specimen corroborates this assertion (see Figure 5e–h). Finally, on the other hand, the isolated nanoparticles in the Bi_*x*_Ti_*y*_O_*z*_/MOF-In_2_S_3_/CdIn_2_S_4_ specimen (Figure 5c,d) are formed by the different bismuth titanates indexed by XRD, with the general formula of Bi_*x*_Ti_*y*_O_*z*_.

The third clear particle morphology is 0D nanoparticles and nanorods (clearly observed in Figure 4e,f, respectively. Attending to the XEDS mapping studies (Figure S3) these particles are composed of Bi, Ti, and O, suggesting they are the bismuth titanates determined by XRD. The composition exposed in Table 1 and determined from areas 1 and 4 in Figure S3 corroborated this assertion. However, the composition calculated did not match with the three bismuth compounds indexed by XRD, that is, the Bi_2_O_3_, the Bi_12_TiO_20,_ and the Bi_4_Ti_3_O_12_. This aspect suggests that these particles are in fact mixed nanoparticles of these three compounds. In this sense, regarding the Ti micrograph for Figure S3, it was detected some nanoparticles in the absence of Ti and the presence of Bi and O, corresponding to Bi_2_O_3_ (marked with a white circle).

The really nanosized groups of particles did not allow for carrying out accurate point EDS for each particle. Besides, Bi_2_O_3_ was also detected as submicrometric particles, as can be observed in Figure 4g, in the zones marked as area 3 and area 4 in Figure S3. The EDS composition determined for those areas (see Table 1) now matched clearly with the Bi_2_O_3_ indexed previously by XRD. Finally, analogous to the submicrometric Bi_2_O_3_, submicrometric bismuth titanates were also found, as can be corroborated in Figure S4 (marked with white circles). Due to interferences with the In_2_O_3_@MOF-53 rods, quantification by EDS was not determined.

### BET Analysis

**Figure 6:**
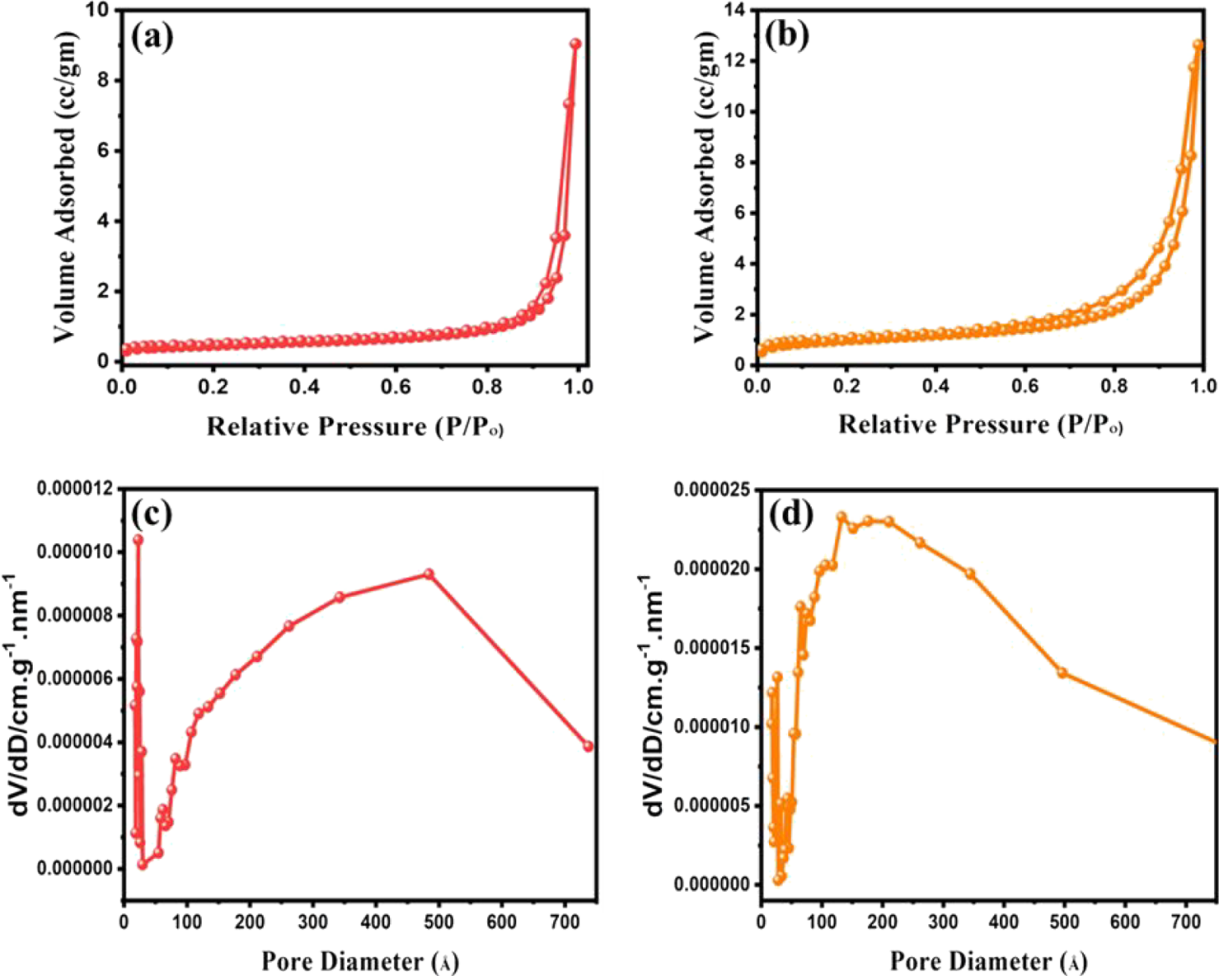
N_2_ adsorption–desorption isotherms and pore size distribution of Bi_*x*_Ti_*y*_O_*z*_ (a and c) and Bi_*x*_Ti_*y*_O_*z*_/MOF-In_2_S_3_/CdIn_2_S_4_ (b and d).

The surface area of bare Bi_*x*_Ti_*y*_O_*z*_ and Bi_*x*_Ti_*y*_O_*z*_/MOF-In_2_S_3_/CdIn_2_S_4_ was determined, and N_2_ adsorption and desorption isotherms of Bi_*x*_Ti_*y*_O_*z*_ and Bi_*x*_Ti_*y*_O_*z*_/MOF-In_2_S_3_/CdIn_2_S_4_ are shown in Figure 6 together with pore size distribution. A type II hysteresis loop can be seen in the isotherms of Bi_*x*_Ti_*y*_O_*z*_ and Bi_*x*_Ti_*y*_O_*z*_/MOF-In_2_S_3_/CdIn_2_S_4_, and their corresponding BET surfaces, pore volumes, and average pore diameters are shown in Table S1. Bi_*x*_Ti_*y*_O_*z*_/MOF-In_2_S_3_/CdIn_2_S_4_ has a larger BET surface area (3.5302 m^2^g^–1^) than that of bare Bi_*x*_Ti_*y*_O_*z*_ (1.6171 m^2^g^–1^). Additionally, Bi_*x*_Ti_*y*_O_*z*_/MOF-In_2_S_3_/CdIn_2_S_4_ has a greater pore volume than pure Bi_*x*_Ti_*y*_O_*z*_.

### XPS Analysis

**Figure 7:**
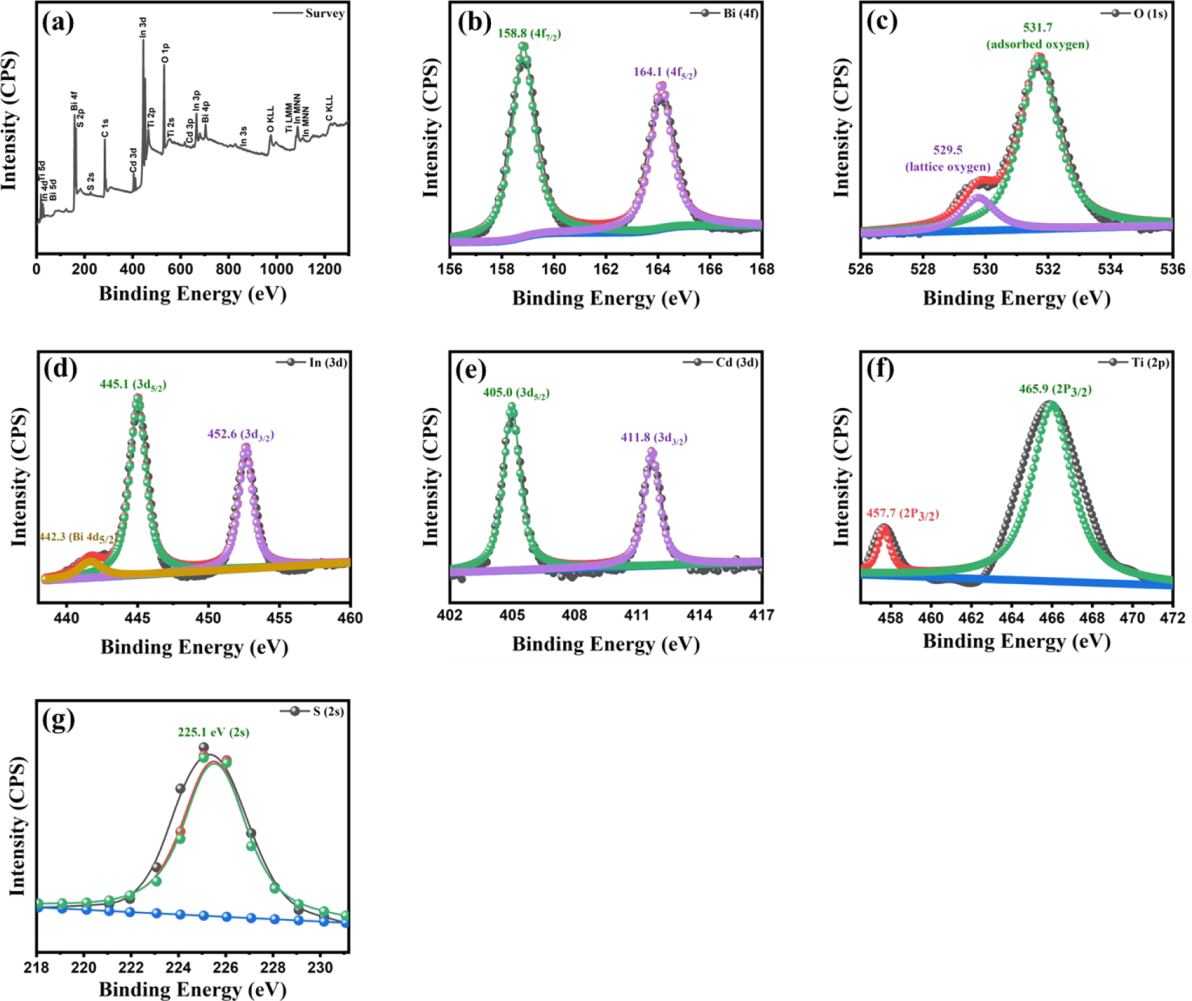
XPS of Bi_*x*_Ti_*y*_O_*z*_/MOF-In_2_S_3_/CdIn_2_S_4_, (a) survey spectrum, (b) Bi 4f, (c) O 1s, (d) In 3d, (e) Cd 3d, (f) Ti 2p, and (g) S 2s.

The surface chemical states of sample Bi_*x*_Ti_*y*_O_*z*_/MOF-In_2_S_3_/CdIn_2_S_4_ were determined by XPS survey spectra, and high-resolution XPS spectra of Bi 4f, O 1s, In 3d, Cd 3d, Ti 2p, and S 2S are shown in Figure 7a–g, respectively. The survey spectrum (Figure 7a) corroborated the existence of Bi, O, In, Cd, Ti, and S elements in the composite. The Bi 4f spectrum (Figure 7b) showed binding energy peaks at 158.8 and 164.1 eV, which were attributed to the 4f_7/2_ and 4f_5/2_ signals having a splitting energy of 5.3 eV. According to the literature, these peaks suggest that Bi is in the 3^+^ state.^54^ The O 1s spectrum in Figure 7c showed two overlapped peaks at 529.5 and 533.7 eV. At a lower binning energy, the peak can be assigned to the metal–oxygen bonds in the bismuth oxide. However, the peak at 531.7 eV could be ascribed to the hydroxyl groups and organic compound present in the MOF.^55,56^

The In spectrum (Figure 7d) showed peaks at 445.1 (3d_5/2_) and 452.6 (3d_3/2_), which can be assigned to the In_2_S_3_ compound^57^ with a splitting energy of 7.5 eV. The peak at 442.3 eV found after the peak deconvoluted was ascribed to the Bi 4d_5/2_ signal of the Bi–S bonds. Figure 7e showed the Cd spectrum with two main peaks at 405.0 (3d_5/2_) and 411.8 (3d_3/2_) having 6.8 eV of peak splitting energy, previously described in the literature for the CdIn_2_S_4_ compound.^58^ In fact, CdIn_2_S_4_ peaks are located at lower binning energies due to the presence of Ti atoms.^59^ The Spectrum of Ti 2p (Figure 7f) showed peaks at 457.7 and 465.9 eV, lower binding energy peak could correspond to Ti 2p_3/2_ at the Ti^3+^ state,^60^ although some differences were found most likely due to the influence of Bi atoms in the existing Bi_12_TiO_20_ phase obtained by the XRD characterization. According to Dongfang Hou,^61^ the peak at 465.9 eV can be caused by the overlapping of peaks Ti 2p_1/2_ and Bi 4d_3/2_ showing a broad bump to higher binding energies. However, due to the small amount of Ti atoms in the sample, the deconvolution peak processing analysis cannot be applied correctly. To identify the presence of sulfur, the S 2s spectrum was used due to the overlapping of the S 2p peaks with Bi 4f. The spectrum in Figure 7g showed a peak at 225.1 eV in agreement with the literature.^62^

### H_2_ Evaluation Activity

**Figure 8:**
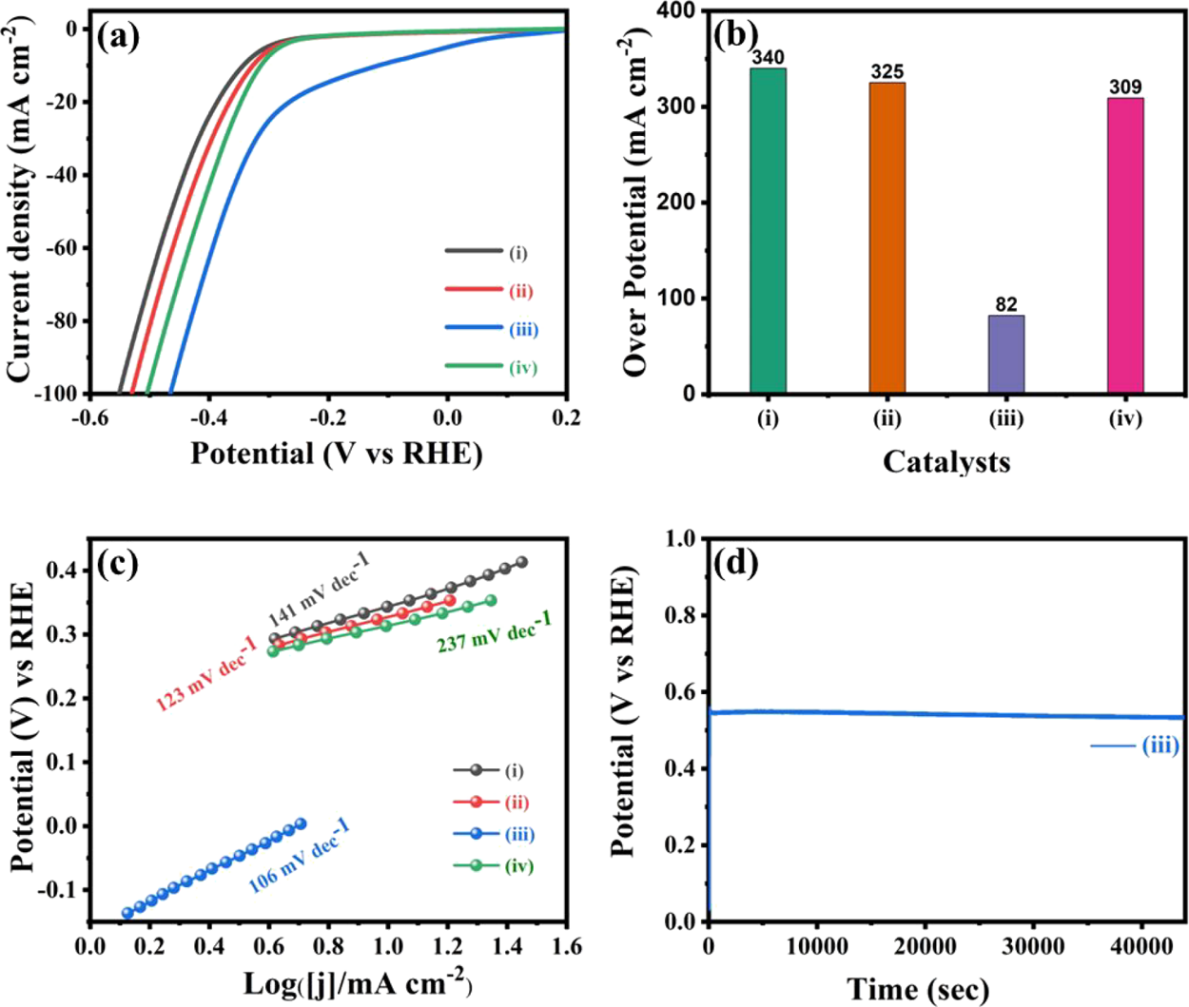
(a) Linear sweep voltammetry (LSV) curves, (b) overpotential, (c) Tafel polarization curves, (d) chronopotentiometric curves for 12 h, of the electrodes in 1.0 M KOH solution at a 10 mV s^–1^ scan rate; (i) MOF-In_2_S_3_, (ii) Bi_*x*_Ti_*y*_O_*z*_/MOF-In_2_S_3_, (iii) Bi_*x*_Ti_*y*_O_*z*_/MOF-In_2_S_3_/CdIn_2_S_4_, and (iv) MOF-In_2_S_3_/CdIn_2_S_4_.

The electrochemical water splitting efficiency of the synthesized Bi_*x*_Ti_*y*_O_*z*_/MOF-In_2_S_3_/CdIn_2_S_4_/NiF electrodes along with those of MOF-In_2_S_3_/NiF, Bi_*x*_Ti_*y*_O_*z*_/MOF-In_2_S_3_/NiF, and MOF-In_2_S_3_/CdIn_2_S_4_/NiF electrodes was also evaluated in a 1 M KOH electrolyte solution using these electrodes with an electrochemical system (Figure 8). The Bi_*x*_Ti_*y*_O_*z*_/MOF-In_2_S_3_/CdIn_2_S_4_/NiF electrode has little significance when compared with MOF-In_2_S_3_/NiF, Bi_*x*_Ti_*y*_O_*z*_/MOF-In_2_S_3_/NiF, and MOF-In_2_S_3_/CdIn_2_S_4_/NiF electrodes, and the corresponding overpotentials are 82, 340, 325, and 309 mV, respectively, as shown in Figure 8a,b. Among the synthesized electrodes, the Bi_*x*_Ti_*y*_O_*z*_ /MOF-In_2_S_3_/CdIn_2_S_4_/NiF electrode has low overpotential (82 mV). As revealed by the overpotentials obtained at 10 mA cm^–2^, the hydrogen evaluation reaction (HER) efficiency improved when Bi_*x*_Ti_*y*_O_*z*_ was incorporated into CdIn_2_S_4_/NiF. Moreover, the synergistic coupling effect of the electron transfer lowers the Gibbs free energy of proton absorption–desorption, responsible for improved HER activities. The Tafel plots obtained from the LSV polarization curves provided valuable insights into the electrochemical water splitting and the properties of the electrocatalyst. The lower Tafel plot indicates a fast increase in the current density relative to the rapid electrochemical water splitting and faster HER kinetics. The appropriate Tafel slopes for the Bi_*x*_Ti_*y*_O_*z*_/MOF-In_2_S_3_/CdIn_2_S4/NiF, MOF-In_2_S_3_/NiF, Bi_*x*_Ti_*y*_O_*z*_/MOF-In_2_S_3_/NiF, and MOF-In_2_S_3_/CdIn_2_S_4_/NiF electrodes for the hydrogen evolution reaction are 106.0, 141.0, 123.0, and 237 mV dec^–1^, respectively (Figure 8c). However, the lowest Tafel slope is obtained for the Bi_*x*_Ti_*y*_O_*z*_/MOF-In_2_S_3_/CdIn_2_S_4_/NiF electrocatalyst because of the synergetic effect of the composite electrocatalyst. Therefore, the Bi_*x*_Ti_*y*_O_*z*_/MOF-In_2_S_3_/CdIn_2_S_4_/NiF composite enhanced the electrocatalytic HER and reaction kinetics performance. The chronopotentiometry analysis performed at a current density of 10 mA cm^–2^ in Figure 8d exhibited the excellent efficiency of the electrocatalyst without any significant loss even after 45000 s of the test. Furthermore, the long-term stability of the as-fabricated catalyst under continuous HER conditions is the most important criterion for potential applications. Therefore, the long-term stability of the efficient Bi_*x*_Ti_*y*_O_*z*_ /MOF-In_2_S_3_/CdIn_2_S_4_/NiF composite catalyst was tested for 1000 CV cycles in the same 1 M KOH electrolyte solution. The initial and 1000-cycle LSV curves for the Bi_*x*_Ti_*y*_O_*z*_/MOF-In_2_S_3_/CdIn_2_S_4_/NiF catalyst are shown in Figure S5. The small overpotential range at the same low current density (10 mA/cm^–1^) indicates the excellent electrochemical stability and durability of the Bi_*x*_Ti_*y*_O_*z*_/MOF-In_2_S_3_/CdIn_2_S_4_/NiF composite catalyst in terms of efficient HER activity.

### TC degradation and Its Mechanism under Solar Light and Literature Comparison

TC is the most fundamental chemical in the TC family and has been utilized extensively throughout human history. When TC usage becomes excessive, this may also result in major health issues. Additionally, it is difficult to eliminate or downgrade the surplus TC. The degradation of TC was conducted under solar light (direct sunlight) to verify the activity of the synthesized materials. The findings are shown in Figure S6a,b, which unmistakably demonstrates that as the irradiation time increases, the degradation of TC is likewise increased with catalysts. When Bi_*x*_Ti_*y*_O_*z*_ was used for TC degradation under solar light, the time of irradiation increased, and the percentage of degradation also increased to 62.5% up to 120 min irradiation; with the further increase in degradation time, there was no enhancement in the degradation efficiency up to 240 min. Bi_*x*_Ti_*y*_O_*z*_/MOF-In_2_S_3_ and Bi_*x*_Ti_*y*_O_*z*_/MOF-In_2_S_3_/CdIn_2_S_4_ were used for the degradation, and the TC concentration was decreased with respect to time and showed 66.4 and 64.1% of degradation, respectively, at 240 min. Under the same conditions, MOF-In_2_S_3_ and MOF-In_2_S_3_/CdIn_2_S_4_ showed 25.9 and 37.4% degradation, respectively. The corresponding kinetics data is provided in Figure S6c. The *pseudo-*first-order kinetics values for Bi_*x*_Ti_*y*_O_*z*_, MOF-In_2_S_3_, Bi_*x*_Ti_*y*_O_*z*_/MOF-In_2_S_3_, Bi_*x*_Ti_*y*_O_*z*_/MOF-In_2_S_3_/CdIn_2_S_4_, and MOF-In_2_S_3_/CdIn_2_S_4_ are 0.00385, 0.00119, 0.00447, 0.00415, and 0.00175 min^–1^, respectively.

Based on the energy levels of these components,^63,64^ an appropriate process for the development of heterostructure among MOF-In_2_S_3_, Bi_*x*_Ti_*y*_O_*z*_, and CdIn_2_S_4_ is provided and the possible TC degradation mechanism under solar light is proposed (Scheme S1). Because MOF-In_2_S_3_ had a CB position that is more negative than that of Bi_*x*_Ti_*y*_O_*z*_, the excited electrons would go from MOF-In_2_S_3_ to Bi_*x*_Ti_*y*_O_*z*_. It was anticipated that h^+^ in the VB of CdIn_2_S_4_ and the accumulated e^–^ in the CB of Bi_*x*_Ti_*y*_O_*z*_ would combine, separating the e^–^ in the CB of CdIn_2_S_4_ and the h^+^ in the VB of Bi_*x*_Ti_*y*_O_*z*_ and improving charge separation efficiency. Highly reactive oxygen species (ROS) are formed when holes and electrons react with water and dissolved oxygen. Both species are quite effective at degrading TC. Table S2 lists the literature comparison with Bi based/modified photocatalysts for TC degradation. The Bi_*x*_Ti_*y*_O_*z*_/MOF-In_2_S_3_/CdIn_2_S_4_ photocatalyst efficiently degrades the TC under direct solar light despite the different optimization techniques.

## Conclusions

For the first time, we use a step-by-step process to design and produce Bi_2_O_3_/bismuth titanates modified with the MOF-In_2_S_3_/CdIn_2_S_4_ materials. The precise structural elucidation and phase development of mixed composite phases were thoroughly investigated. From the XRD, for the specimen, Bi_*x*_Ti_*y*_O_*z*_ showed the presence of a majority phase bismuth oxide (Bi_2_O_3_), with a monoclinic structure and *P*21/*c* SGS along with two different bismuth titanates Bi_12_TiO_20_ and the Bi_4_Ti_3_O_12._ Finally, the other two specimens were developed based on a mixture of Bi_*x*_Ti_*y*_O_*z*_ and MOF-In_2_S_3_ (specimen Bi_*x*_Ti_*y*_O_*z*_/MOF-In_2_S_3_) and the mixture of Bi_*x*_Ti_*y*_O_*z*_ and MOF-In_2_S_3_/CdIn_2_S_4_ (specimen Bi_*x*_Ti_*y*_O_*z*_/MOF-In_2_S_3_/CdIn_2_S_4_). The PL intensity of the Bi_*x*_Ti_*y*_O_*z*_/MOF-In_2_S_3_/CdIn_2_S_4_ specimen is low compared with those of other components. The TEM study was carried out on the Bi_*x*_Ti_*y*_O_*z*_/MOF-In_2_S_3_/CdIn_2_S_4_ specimen, and it confirmed that this composition contains a mixture of the specimens Bi_*x*_Ti_*y*_O_*z*_ and MOF-In_2_S_3_/CdIn_2_S_4_. Thus, the introduction of Cd in Bi_*x*_Ti_*y*_O_*z*_/MOF-In_2_S_3_/CdIn_2_S_4_ caused the transformation of some In_2_S_3_ by In_2_O_3_ because of the excess amount of S required, and the remaining excess In^3+^ cations reacted with oxygen to form nanoembedded particles of In_2_O_3_; this was confirmed by HRTEM measurements of the Bi_*x*_Ti_*y*_O_*z*_/MOF-In_2_S_3_ specimen. From XPS, CdIn_2_S_4_ peaks are located at lower binning energies due to the presence of Ti atoms. The HER efficiency improved when Bi_*x*_Ti_*y*_O_*z*_ was incorporated into CdIn_2_S_4_/NiF. Moreover, the synergistic coupling effect of the electron transfer lowers the Gibbs free energy of proton absorption–desorption and is responsible for improved HER activities. The appropriate Tafel slopes for the Bi_*x*_Ti_*y*_O_*z*_/MOF-In_2_S_3_/CdIn_2_S_4_/NiF, MOF-In_2_S_3_/NiF, Bi_*x*_Ti_*y*_O_*z*_/MOF-In_2_S_3_/NiF, and MOF-In_2_S_3_/CdIn_2_S_4_/NiF electrodes for the hydrogen evolution reaction are 106.0, 141.0, 123.0, and 237 mV dec^–1^, respectively. The prepared composites were effectively utilized for TC degradation under solar light. Improved electrocatalytic activity of the material and photocatalytic activity under direct sunlight is favorable in the development of catalytic support for energy and environmental applications.

## Supporting Information Available

FE-SEM images of Bi_*x*_Ti_*y*_O_*z*_, MOF-In_2_S_3_, and Bi_*x*_Ti_*y*_O_*z*_/MOF-In_2_S_3_/CdIn_2_S_4_, FE-SEM images of for Bi_*x*_Ti_*y*_O_*z*_/MOF-In_2_S_3_, and MOF-In_2_S_3_/CdIn_2_S_4_, mapping STEM images for the Bi_*x*_Ti_*y*_O_*z*_/MOF-In_2_S_3_/CdIn_2_S_4_ material, polarization curves of the efficient Bi_*x*_Ti_*y*_O_*z*_/MOF-In_2_S_3_/CdIn_2_S_4_/NiF for the first and 1000th cycles, photodegradability of TC under direct solar light, mechanism of TC degradation by Bi_*x*_Ti_*y*_O_*z*_/MOF-In_2_S_3_/CdIn_2_S_4_ under solar light, surface properties of the Bi_*x*_Ti_*y*_O_*z*_ and Bi_*x*_Ti_*y*_O_*z*_/MOF-In_2_S_3_/CdIn_2_S_4_, and comparison of TC degradation efficiency (PDF)
