# Supplementary material for: Fabrication of Bi2O3/Bismuth Titanates Modified with Metal–Organic Framework-In2S3/CdIn2S4 Materials for Electrocatalytic H2 Production and Its Photoactivity
Source: Langmuir. 2023 Oct 16;39(42):15055–66. doi: 10.1021/acs.langmuir.3c02031 (PMC10601539; doi:10.1021/acs.langmuir.3c02031)
Supplement: Supplementary file 1 — la3c02031_si_001.pdf [file la3c02031_si_001.pdf]

Supporting information's on

Fabrication of Bi_2O_3/Bismuth Titanates Modified with Metal Organic Framework-In_2S_3/$CdIn_2S_4$ Materials for Electrocatalytic H_2 Production and Its Photoactivity

Krishnakumar Balu[1a,g]*, Balakrishna Avula[b], Mani Durai[c1], Sakthivel Kumaravel[d], Ernesto Chicardi*[a], Ranier Sepúlveda[a], Elangovan Erusappan[e], Imran Hasan[f], and Young-Ho Ahn[c]*

[a]Departamento de Ingeniería y Ciencia de los Materiales y del Transporte, E.T.S. de Ingenieros, Universidad de Sevilla, Avda. Camino de los Descubrimientos s/n., 41092 Sevilla, Spain.

[b]Department of Chemistry, Rajeev Gandhi Memorial College of Engineering and Technology (Autonomous), Nandyal-518501, India

[c]Environmental Science and Engineering Laboratory, Department of Civil Engineering, Yeungnam University, Gyeongsan, 38541, Republic of Korea.

[d]Department of Environmental Engineering, Korea Maritime and Ocean University, Busan 49112, Republic of Korea

[e]Department of Applied Science and Technology, Anna University, Chennai 600025, India

[f]Department of Chemistry, College of Science, King Saud University, Riyadh-11451, Saudi Arabia.

[g]Department of Chemistry, Saveetha School of Engineering, Saveetha Institute of Medical and Technical Sciences, Saveetha University, Chennai- 602105, Tamil Nadu, India.

[1] These authors contributed equally to this work

*Corresponding authors:

Dr. Krishnakumar Balu

Departamento de Ingeniería y Ciencia de los Materiales y del Transporte,

E.T.S. de Ingenieros, Universidad de Sevilla,

Avda. Camino de los Descubrimientos s/n., 41092 Sevilla,

Spain.

E-mail: kbalu@us.es (Dr. Krishnakumar Balu)

E-mail: echicardi@us.es (Dr. E. Chicardi)

E-mail: yhahn@ynu.ac.kr (Dr. Young-Ho Ahn)

Table of Contents

S.No	Details	Page number
1	**Figure S1**. FE-SEM images of for $Bi_xTi_yO_z$(a), MOF-In_2S_3(b&c) and $Bi_xTi_yO_z$/MOF-In_2S_3/$CdIn_2S_4$ (d-f)	3
2	**Figure S2**. FE-SEM images of for $Bi_xTi_yO_z$/MOF-In_2S_3 (a-c), and MOF-In_2S_3/$CdIn_2S_4$(d-f)	3
3	**Figure S3.** Mapping STEM images for the $Bi_xTi_yO_z$/MOF-In_2S_3/$CdIn_2S_4$ material, showed the bismuth titanates nature of the nanoparticles and nanorods ($Bi_xTi_yO_z$) (area #1 and #4) and the $CdIn_2S_4$ nature of the flowers (area #2 and #3). Colours in mapping: pink = Cd; light blue = In; orange = S; green = Bi; red = Ti; and yellow = O	4
4	**Figure S4.** Mapping STEM images for the $Bi_xTi_yO_z$/MOF-In_2S_3/$CdIn_2S_4$ material, showed the submicrometric bismuth titanates nature (Bi_xTiO_y) close to the In_2O_3@MOF-53 rods. Again, the $CdIn_2S_4$ nature of the flowers (area #1) can be corroborated and the presence of In and O in the area #2, corresponding to the In_2O_3@MOF-53 rods. Colours in mapping: pink = Cd; light blue = In; orange = S; green = Bi; red = Ti; and yellow = O	5
5	**Figure S5.** Polarization curves of the efficient $Bi_xTi_yO_z$/MOF-In_2S_3/$CdIn_2S_4$/NiF catalyst for the first and 1000th cycles	6
6	**Figure S6.** Photodegradability of TC under direct solar light (a) Degradation of TC with respect to time, and (b) corresponding C_t/C_0 values, (c) corresponding kinetics data , (i) $Bi_xTi_yO_z$, (ii) MOF-In_2S_3, (iii) $Bi_xTi_yO_z$/MOF-In_2S_3, (iv) $Bi_xTi_yO_z$ /MOF-In_2S_3/$CdIn_2S_4$ and (v) MOF-In_2S_3/$CdIn_2S_4$, [TC] = 20 ppm/100 mL; catalyst amount = 20 mg/ 100 mL; Solar light = 95 mW/cm^2	7
7	**Scheme S1.** Mechanism of TC degradation by $Bi_xTi_yO_z$ /MOF-In_2S_3/$CdIn_2S_4$ under solar light	8
8	**Table S1.** Surface properties of the $Bi_xTi_yO_z$ and $Bi_xTi_yO_z$/MOF-In_2S_3/$CdIn_2S_4$	9
9	**Table S2**. Comparison of TC degradation efficiency of the $Bi_xTi_yO_z$/MOF-In_2S_3/$CdIn_2S_4$ composite with Bi_2O_3 based catalysts	10
10	**References**	11

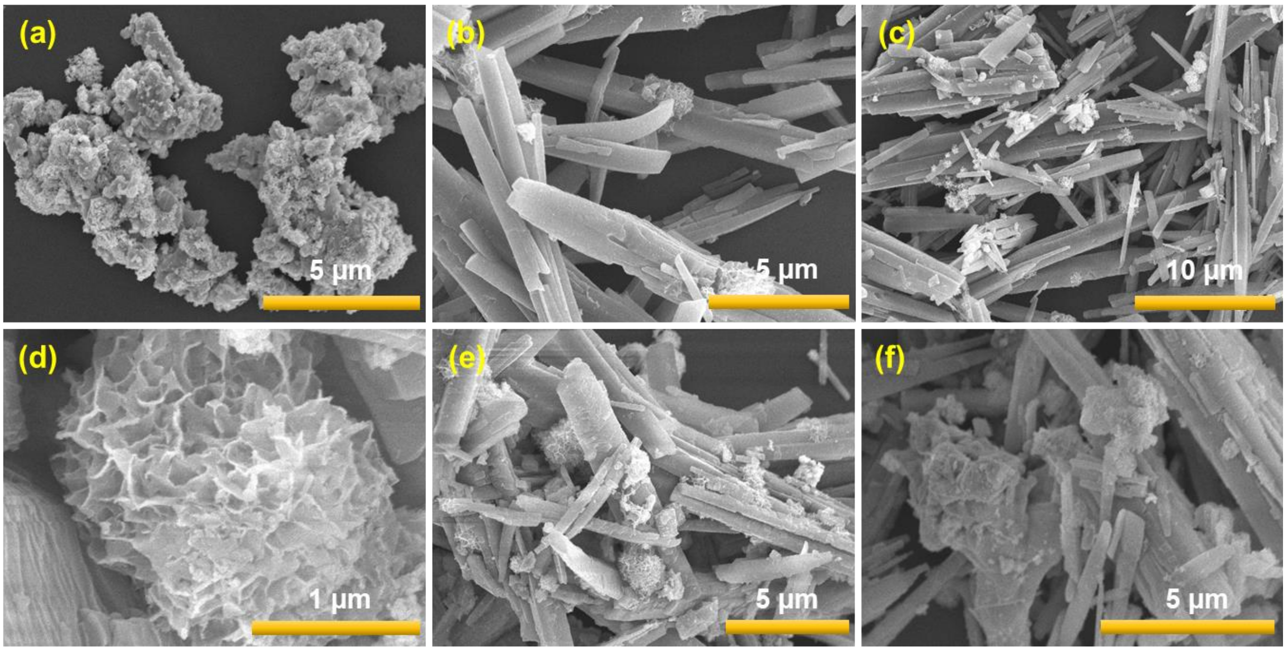

Figure S1. FE-SEM images of for $Bi_xTi_yO_z$(a), MOF-In_2S_3(b&c) and $Bi_xTi_yO_z$/MOF-In_2S_3/$CdIn_2S_4$ (d-f)

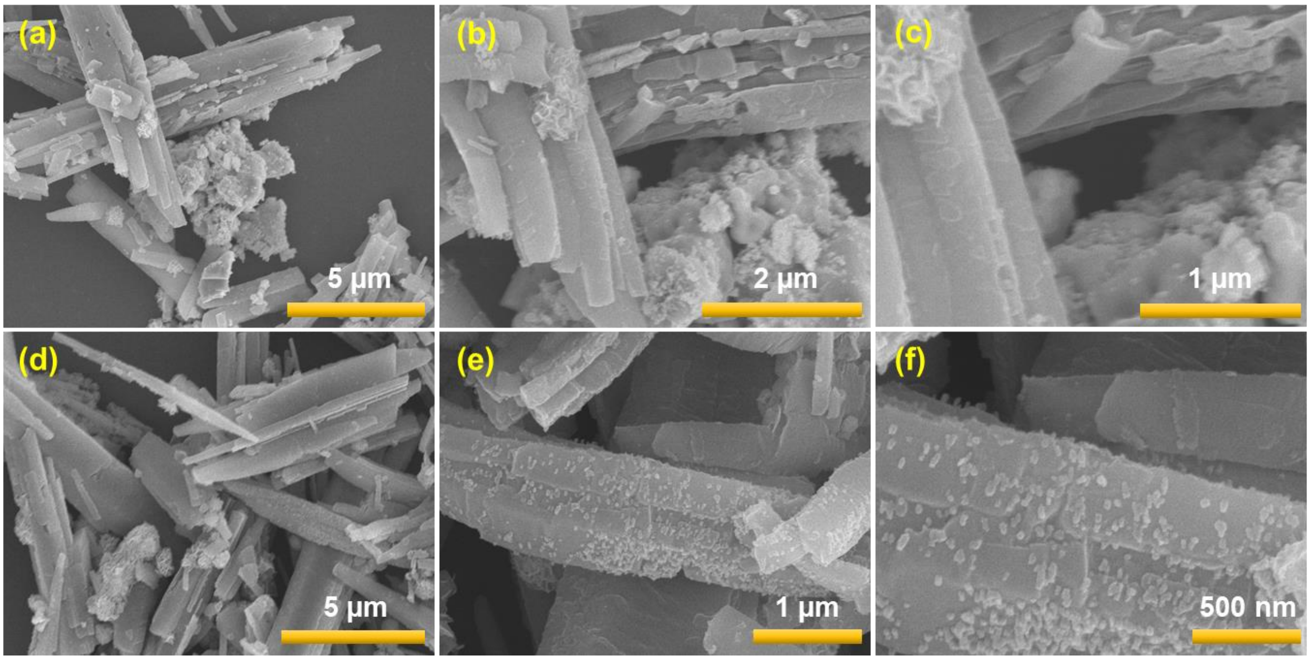

Figure S2. FE-SEM images of for $Bi_xTi_yO_z$/MOF-In_2S_3 (a-c), and MOF-In_2S_3/$CdIn_2S_4$(d-f)

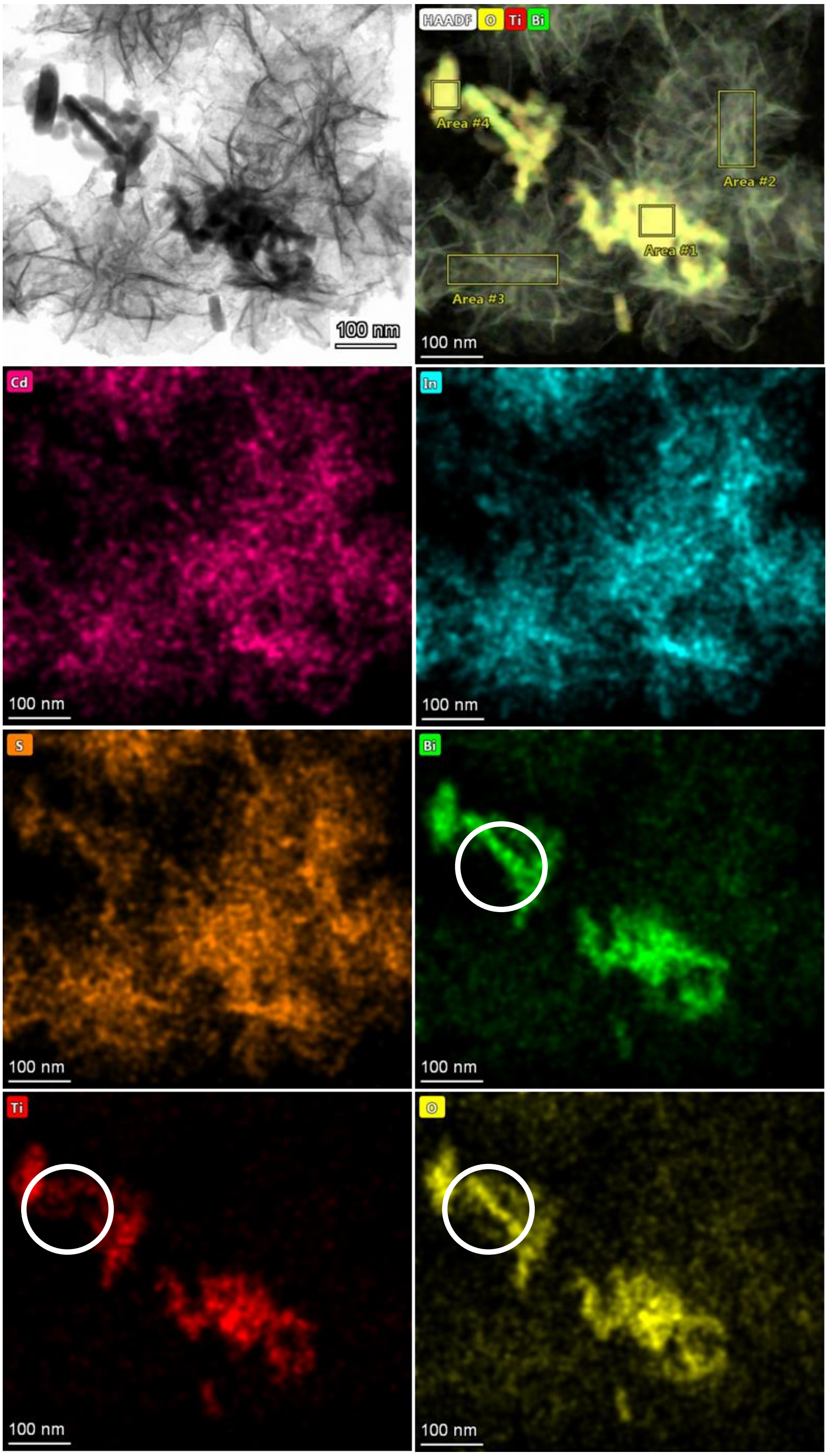

Figure S3. Mapping STEM images for the $Bi_xTi_yO_z$/MOF-In_2S_3/$CdIn_2S_4$ material, showed the bismuth titanates nature of the nanoparticles and nanorods ($Bi_xTi_yO_z$) (area #1 and #4) and the $CdIn_2S_4$ nature of the flowers (area #2 and #3). Colours in mapping: pink = Cd; light blue = In; orange = S; green = Bi; red = Ti; and yellow = O

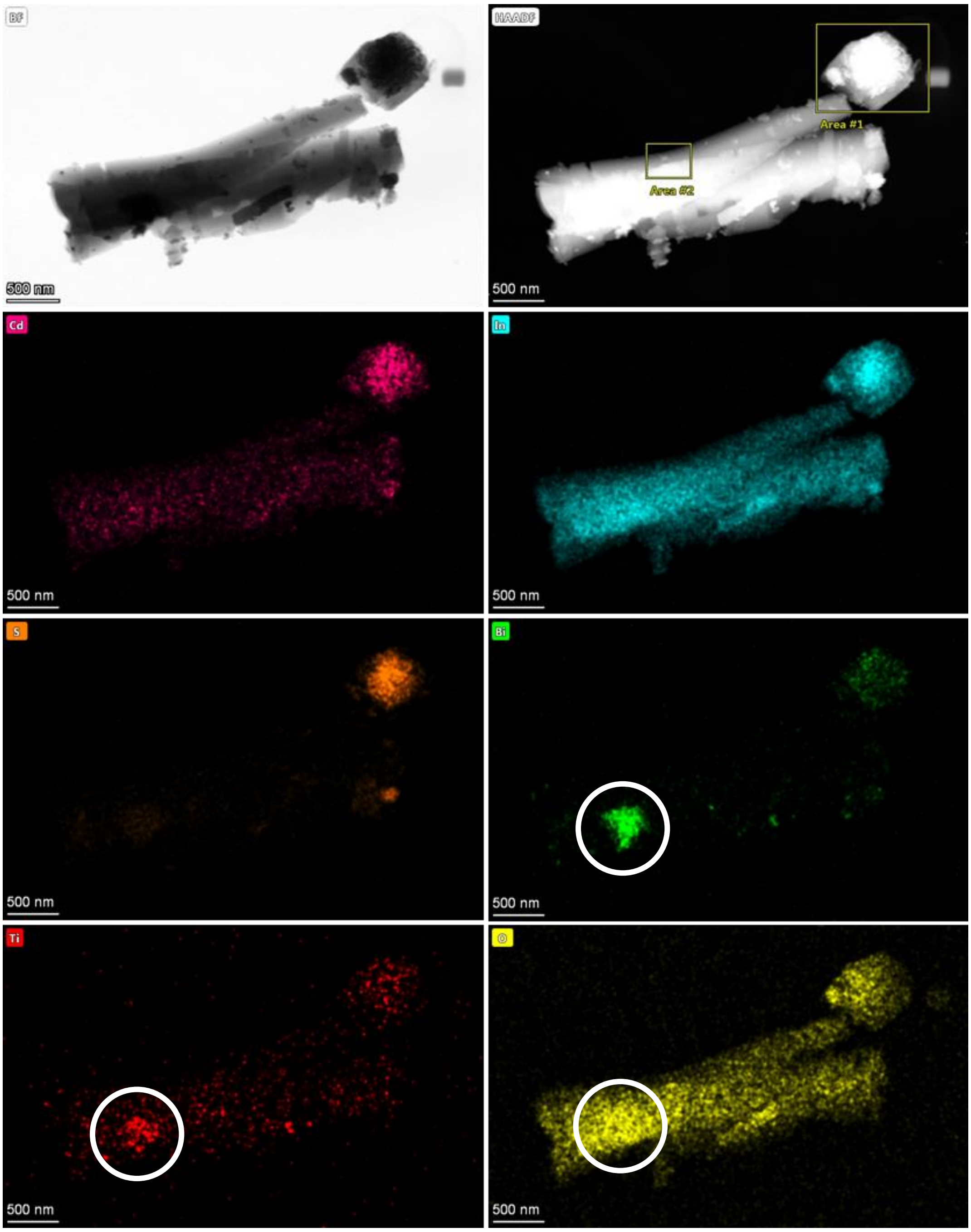

Figure S4. Mapping STEM images for the $Bi_xTi_yO_z$/MOF-In_2S_3/$CdIn_2S_4$ material, showed the submicrometric bismuth titanates nature (Bi_xTiO_y) close to the In_2O_3@MOF-53 rods. Again, the $CdIn_2S_4$ nature of the flowers (area #1) can be corroborated and the presence of In and O in the area #2, corresponding to the In_2O_3@MOF-53 rods. Colours in mapping: pink = Cd; light blue = In; orange = S; green = Bi; red = Ti; and yellow = O

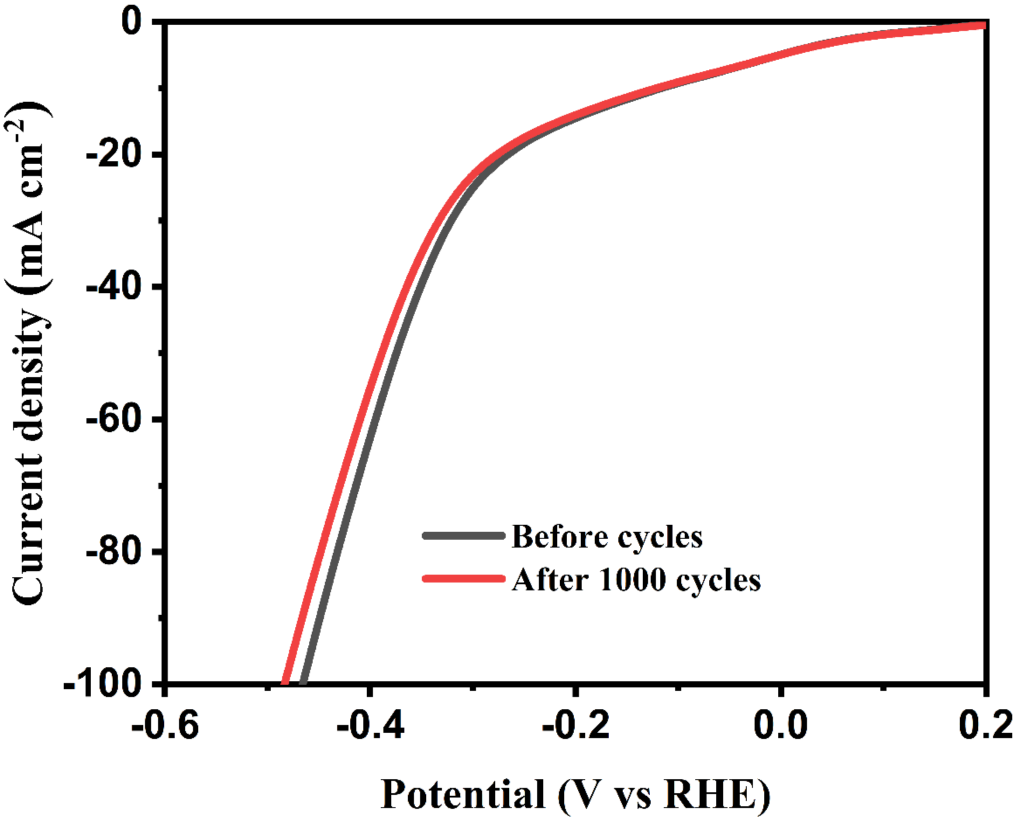

Figure S5. Polarization curves of the efficient $Bi_xTi_yO_z$/MOF-In_2S_3/$CdIn_2S_4$/NiF catalyst for the first and 1000th cycles

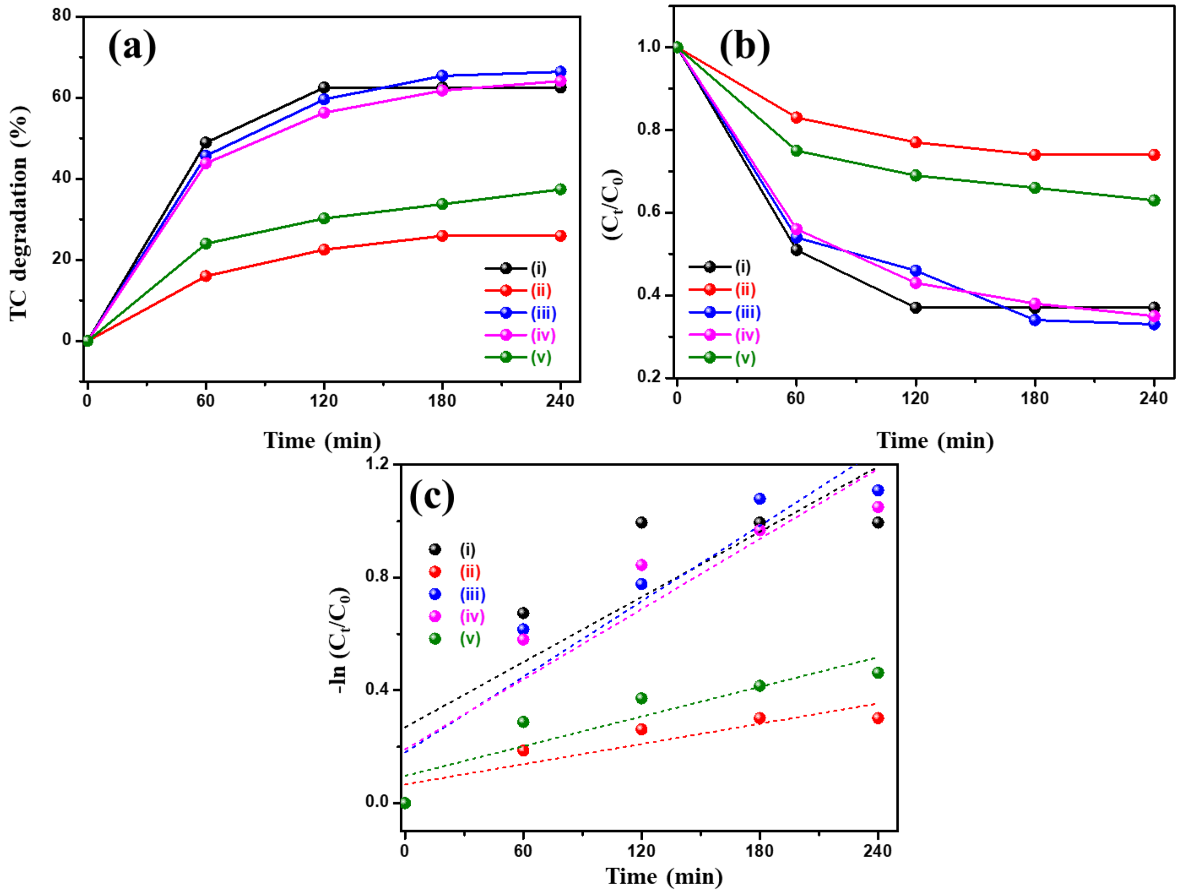

Figure S6. Photodegradability of TC under direct solar light (a) Degradation of TC with respect to time, and (b) corresponding C_t/C_0 values, (c) corresponding kinetics data , (i) $Bi_xTi_yO_z$, (ii) MOF-In_2S_3, (iii) $Bi_xTi_yO_z$/MOF-In_2S_3, (iv) $Bi_xTi_yO_z$ /MOF-In_2S_3/$CdIn_2S_4$ and (v) MOF-In_2S_3/$CdIn_2S_4$, [TC] = 20 ppm/100 mL; catalyst amount = 20 mg/ 100 mL; Solar light = 95 mW/cm^2

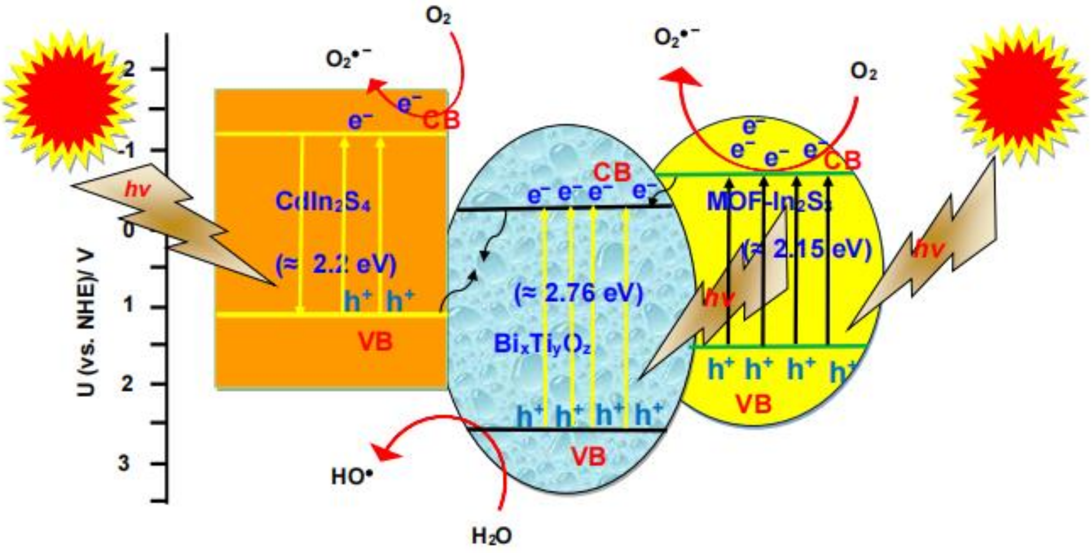

$$TC + HO^{\cdot} \longrightarrow \text{Mineral acids} + CO_2\uparrow + H_2O$$

$$TC + O_2^{\cdot-} \longrightarrow \text{Mineral acids} + CO_2\uparrow + H_2O$$

Scheme S1. Mechanism of TC degradation by $Bi_xTi_yO_z$ /MOF-In_2S_3/$CdIn_2S_4$ under solar light

Table S1. Surface properties of the $Bi_xTi_yO_z$ and $Bi_xTi_yO_z$/MOF-In_2S_3/$CdIn_2S_4$

**Properties**	$Bi_xTi_yO_z$	$Bi_xTi_yO_z$/MOF-In_2S_3/$CdIn_2S_4$
BET surface area	1.6171 (m^2 g^{-1})	3.5302 (m^2 g^{-1})
BJH Desorption cumulative volume of pores	0.013759 (cm^3 g^{-1})	0.019063 (cm^3 g^{-1})
BJH Adsorption average pore diameter (4V/A)	452.046 Å	300.037 Å

Table S2. Comparison of TC degradation efficiency of the $Bi_xTi_yO_z$/MOF-In_2S_3/$CdIn_2S_4$ composite with Bi_2O_3 based catalysts

S.No	Catalysts[a]	Light source	Concentration	Catalyst amount	%Degradation/ time (min)	Ref.
1	Bi_2O_3/Bi/$ZnIn_2S_4$	500 W, Xenon lamp (λ > 420 nm)	10 ppm	50 mg/50 mL	96.5/60	[S1]
2	Bi_2O_3/biochar/g-C_3N_4	500 W, Xenon lamp (λ = 420 nm)	20 ppm	50 mg/50 mL	86.7/30	[S2]
3	Bi_2S_3/Bi_2O_3	Xenon light, 300 W	50 ppm	200 mg /200 mL	≈100/90	[S3]
4	Tubular g-C_3N_4/ Bi_2O_3	Xenon light, 300 W with 420 nm cutoff filter	10 ppm	50 mg/100mL	94/100	[S4]
5	β-Bi_2O_3/$Bi_2O_2CO_3$	500 W, Xenon light	20 ppm	50 mg/50 mL	95.3/60	[S5]
6	$Bi_xTi_yO_z$/MOF-In_2S_3	Direct solar light 95 mW/cm^2	20 ppm	20 mg/ 100 mL	66.4/240	This work
7	$Bi_xTi_yO_z$/MOF-In_2S_3/$CdIn_2S_4$	Direct solar light 95 mW/cm^2	20 ppm	20 mg/ 100 mL	64.1/240	This work

[a]ZIF-67=Cobalt salt with 2-methylimidazole

From Table S2, the $Bi_xTi_yO_z$/MOF-In_2S_3/$CdIn_2S_4$ photocatalyst efficiently degrades the TC under direct solar light despite the different optimization techniques. We have used less catalytic amount in the process, and the degradation was performed under direct sunlight.

REFERENCES

S1. Luo, J.; Shi, Z.; Meng, J.; Li, F.; Li, T.; Zhang, M.; Greco, R.; Cao, W. Z-scheme Bi_2O_3/Bi/$ZnIn_2S_4$ photocatalyst for enhancing the removal performance of Cr(VI), 2,4-dinitrophenol and tetracycline, *J. Ind. Eng. Chem.* **2023**, 124, 250–262.

S2. Huang, L.; Liu, H.; Wang, Y.; Zhang, T.C.; Yuan, S. Construction of ternary Bi_2O_3/biochar/g-C_3N_4 heterojunction to accelerate photoinduced carrier separation for enhanced tetracycline photodegradation, *Appl. Surf. Sci.,* **2023**, 616, 156509.

S3. Kashmery, H. A.; El-Hout, S.I. Bi_2S_3/Bi_2O_3 nanocomposites as effective photocatalysts for photocatalytic degradation of tetracycline under visible-light exposure, *Opt. Mater.,* **2023**, 135, 113231.

S4. Pei, X.; An, W.; Zhao, H.; He, H.; Fu, Y.; Shen, X. Enhancing visible-light degradation performance of g-C_3N_4 on organic pollutants by constructing heterojunctions via combining tubular g-C_3N_4 with Bi_2O_3 nanosheets, *J. Alloys Compd.,* **2023**, 934, 167928.

S5. Huang, Y.; Zhang, X.; Zheng, F.; Zou, S.; Li, M.; Huang, P.; Zeng, Y. Controlled synthesis of β-Bi_2O_3/$Bi_2O_2CO_3$ hollow microspheres with enhanced photocatalytic degradation of tetracycline under visible light, *Mater. Today Commun,* **2022**, 33, 104304.
